# Potential population-level effectiveness of one-dose HPV vaccination in low-income and middle-income countries: a mathematical modelling analysis

**DOI:** 10.1016/S2468-2667(23)00180-9

**Published:** 2023-09-28

**Authors:** Élodie Bénard, Mélanie Drolet, Jean-François Laprise, Guillaume Gingras, Mark Jit, Marie-Claude Boily, Paul Bloem, Marc Brisson

**Affiliations:** a Département de médecine sociale et préventive, Université Laval, Québec City, QC, Canada; b Centre de recherche du CHU de Québec—Université Laval, Québec City, QC, Canada; c Centre for Mathematical Modelling of Infectious Disease, London School of Hygiene & Tropical Medicine, London, UK; d School of Public Health, University of Hong Kong, Hong Kong Special Administrative Region, China; e MRC Centre for Global Infectious Disease Analysis, School of Public Health, Imperial College London, London, UK; f Life Course and Integration, Essential Programme on Immunization, Department of Immunization, Vaccines and Biologicals, World Health Organization, Geneva, Switzerland

## Abstract

**Background:**

Given the accumulating evidence that one-dose vaccination could provide high and sustained protection against human papillomavirus (HPV) infection and related diseases, we examined the population-level effectiveness and efficiency of one-dose HPV vaccination of girls compared with two-dose vaccination, using mathematical modelling.

**Methods:**

In this mathematical modelling study, we used HPV-ADVISE LMIC, an individual-based transmission-dynamic model independently calibrated to four epidemiologically diverse low-income and middle-income countries (LMICs; India, Nigeria, Uganda, and Viet Nam). We parameterised and calibrated the model using sexual behaviour and epidemiological data identified from international population-based datasets and the literature. All base-case vaccination scenarios start in 2023 with the nonavalent vaccine and assumed 80% vaccination coverage with one or two doses. We assumed that two doses of vaccine provide 100% efficacy against vaccine-type infections and a lifelong duration of protection. We examined a non-inferior vaccination scenario for one dose compared with two doses, pessimistic scenarios of lower one-dose vaccine efficacy (85%) or a shorter duration of protection (ie, 20 or 30 years), and the effectiveness of a mitigation scenario in which schedules would switch from one dose to two doses. We also did sensitivity analyses by varying vaccination coverage. We used three outcomes: the relative reduction in cervical cancer incidence, the number of cervical cancers averted, and the number of vaccine doses needed to prevent one cervical cancer.

**Findings:**

Assuming non-inferior vaccine characteristics for one dose compared with two doses, the model projections show that two-dose or one-dose routine vaccination of girls aged 9 years (with a multi-age cohort vaccination of girls aged 10–14 years) would avert 12·0 million (80% UI 9·5–14·5) cervical cancers in India, 4·7 million (3·4–5·8) in Nigeria, 2·3 million (1·9–2·6) in Uganda, and 0·4 million (0·2–0·5) in Viet Nam over 100 years. Under pessimistic assumptions of lower one-dose efficacy (85%) or a shorter duration of protection (ie, 30 years), one-dose routine vaccination would avert 69% (61–80) to 94% (92–96) of the cervical cancers averted with two-dose routine vaccination. However, when assuming a duration of protection of 20 years, one-dose routine vaccination would avert substantially fewer cervical cancers (ie, 35% [26–44] to 69% [65–71] of the cervical cancers averted with two-dose routine vaccination). A switch from one-dose to two-dose routine vaccination of girls aged 9 years, with a one-dose catch-up of girls aged 10–14 years, 5 years after the start of the vaccination programme, could mitigate potential losses in cervical cancer prevention from a short one-dose duration of protection (averting 92% [83–98] to 99% [97–100]) of the cervical cancers averted with two-dose routine vaccination). One-dose routine vaccination would result in fewer doses needed to prevent one cervical cancer than two-dose routine vaccination, even if the duration of protection is as low as 20 years. Finally, for countries with two-dose routine vaccination, adding one-dose multi-age cohort vaccination in the first year would provide similar benefits as a two-dose multi-age cohort vaccination, and would be more efficient even under the pessimistic assumptions of lower one-dose vaccine efficacy or duration of protection.

**Interpretation:**

One-dose routine vaccination could avert most of the cervical cancers averted with two-dose vaccination while being more efficient, provided the duration of one-dose protection is greater than 20–30 years (depending on the LMIC). The doses saved by introducing one-dose routine vaccination could offer the opportunity to vaccinate girls before they age out of the vaccination window of 9–14 years and, potentially, to vaccinate boys or older age groups.

**Funding:**

Fonds de recherche du Québec—Santé, Digital Research Alliance of Canada, Bill & Melinda Gates Foundation.

**Research in context**

**Evidence before this study**

Although more than 85% of cervical cancers worldwide are detected in low-income and middle-income countries (LMICs), only about 40% of LMICs have introduced human papillomavirus (HPV) vaccination compared with more than 80% of high-income countries (HICs). One-dose vaccination could alleviate the programmatic and financial challenges of two-dose schedules. We searched MEDLINE to identify modelling studies that projected the population-level effectiveness of one-dose HPV vaccination. We searched the database on March 8, 2023, (no date or language restriction) with the following combination of MeSH terms, title, and abstract words: (“single dose” or “one dose”) and (“human papillomavirus vaccine” or “human papillomavirus vaccination” or “hpv vaccine” or “hpv vaccination”) and (“models, theoretical” or “model” or “models” or “modeling study” or “modelling study”). To be eligible, studies had to examine the potential population-level effectiveness of one-dose HPV vaccination in an LMIC. We identified three recent modelling studies that met our criteria. These studies investigated the potential population-level effectiveness of one-dose HPV vaccination in India, Uganda, and China, suggesting favourable outcomes for one-dose vaccination.

**Added value of this study**

We have expanded on these studies by examining the effectiveness of one-dose HPV vaccination in four LMICs with differences in sexual activity and cervical cancer burden and by exploring a large range of vaccine characteristics and vaccination programme assumptions. In this modelling study, we examined the effectiveness of one-dose schedules for different LMIC contexts: countries introducing one-dose vaccination for the first time with high and low vaccination coverage and countries that would switch from a two-dose to a one-dose schedule. We showed that, for four LMICs (India, Nigeria, Uganda, and Viet Nam), if vaccine efficacy is greater than 85% or lasts longer than 20–30 years (depending on the LMIC), one-dose vaccination could substantially reduce cervical cancer incidence, avert most of the cervical cancers averted with two-dose vaccination, and be more efficient than two-dose vaccination for the different LMIC profiles and vaccination contexts examined. Our results also suggest that, if studies start showing evidence of substantial waning of one-dose protection within the next 5 years, by which time more than 15 years of one-dose immunogenicity and efficacy data will be available, switching to two-dose routine vaccination could mitigate any potential losses in cervical cancer prevention.

**Implications of all the available evidence**

The overall evidence suggests that one-dose routine vaccination could avert most of the cervical cancers averted with two-dose vaccination if the vaccine's duration of protection is greater than 20–30 years, while being more efficient, easier to implement, and less costly. One-dose vaccination could also allow for extension of vaccination programmes to other groups in LMICs such as boys or older women to maximise the population-level effectiveness and reduce inequalities between LMICs and HICs. However, as duration of protection is a key determinant of the population-level effectiveness of one-dose vaccination, we must continue monitoring one-dose vaccine efficacy and immunogenicity over time to rapidly detect any decrease in protection, and implement mitigation strategies if needed.

## Introduction

More than 85% of cervical cancers are detected in low-income and middle-income countries (LMICs).^1^ The disparity in cervical cancer burden is set to increase as, in 2020, only 41% of LMICs had introduced human papillomavirus (HPV) vaccination compared with 88% of high-income countries (HICs).^2^, ^3^ Furthermore, 44% of HICs also vaccinate boys and 81% have introduced multi-age cohort vaccination, either as campaigns or as part of their routine programmes.^3^ As a result, HICs are already seeing important reductions in HPV infections and related diseases, including cervical cancer.^4^, ^5^, ^6^ Register-based studies have shown a decrease of 87% in cervical cancer incidence among women aged 30 years and younger (vaccinated at age ≤14 years) in Denmark and the UK.^4^, ^5^

The introduction of two-dose HPV vaccination in LMICs has been slowed down due to resource constraints, competing health-care priorities, and the COVID-19 pandemic.^7^, ^8^ Furthermore, the COVID-19 pandemic led to decreases in vaccination coverage in countries that had started HPV vaccination before the pandemic, particularly LMICs. Compared with 2019, two-dose vaccination coverage in 2020 decreased by 17 percentage points in low-income countries, 11 percentage points in middle-income countries, and 10 percentage points in HICs.^9^, ^10^ The delayed introduction of HPV vaccination and reduced vaccination coverage in LMICs might result in many cohorts of girls not getting vaccinated as they age out of the primary target group of vaccination of 9–14 years old.^8^, ^11^ If effective, one-dose vaccination could reduce the programmatic and financial challenges of two-dose vaccination, and the doses saved could provide the opportunity to reach girls aged 10–14 years before they age out of the school-based vaccination window through multi-age cohort vaccination.

There is increasing evidence suggesting that one-dose vaccination could provide high and sustained protection against HPV infections and related diseases.^12^, ^13^, ^14^, ^15^, ^16^, ^17^ Results from a large multicentre prospective cohort study of participants of a trial^12^, ^13^ done by the International Agency for Research on Cancer (IARC) in India, who received different numbers of doses for reasons unrelated to their HPV infection risk, showed one-dose vaccine efficacy of 94% against HPV 16 or 18 persistent infection, up to 10 years after vaccination. Results from the CVT trial^14^, ^15^ in Costa Rica also showed similar efficacy of one and three doses of the bivalent vaccine against HPV 16 and 18 infections and stability of antibodies with one dose, up to 16 years after vaccination. An ongoing randomised controlled trial in Kenya^16^, ^17^ also showed a very high one-dose vaccine efficacy (98·6% against HPV 16, 18, 31, 33, 45, 52, and 58 among women aged 15–20 years). Finally, the first results from the DoRIS immunobridging study^18^ indicated that the antibody concentrations among girls aged 9–14 years from sub-Saharan Africa were immunologically non-inferior to the concentrations in women from the IARC India and CVT trials.^12^, ^14^, ^18^

In view of the accumulating evidence on one-dose vaccine efficacy, the WHO Strategic Advisory Group of Experts on Immunization (SAGE) examined whether an off-label permissive one-dose HPV vaccine schedule should be recommended for use in the primary target of girls aged 9–14 years. This Article includes modelling analyses presented to SAGE in April, 2022, to help inform their global HPV vaccination policy recommendations on one-dose HPV vaccination.^19^ Our objective was to examine, using mathematical modelling, the population-level effectiveness and efficiency of one-dose compared with two-dose vaccination of girls for four LMICs (India, Nigeria, Uganda, and Viet Nam) over 100 years, under different assumptions of one-dose efficacy, duration of protection, and vaccination coverage.

## Methods

### Study design

In this mathematic modelling analysis, we used the HPV-ADVISE LMIC model. The model has been previously used to help inform global HPV vaccination policy recommendations and has been extensively described in previous papers.^20^, ^21^ Briefly, HPV-ADVISE LMIC is an individual-based, transmission-dynamic model of HPV infection and disease (appendix 1 pp 1–5). The populations modelled represent the heterosexual population specific to each of the four countries. 18 HPV types are modelled independently (6 and 11, which cause genital warts, and 16, 18, 31, 33, 45, 52, 58, 35, 39, 51, 56, 59, 66, 68, 73, and 82, which can lead to cervical cancer) and each HPV type has its own natural history parameters. The model contains five different integrated modules: demographic characteristics, sexual behaviour and transmission of HPV, natural history of HPV-associated diseases (HPV infection, natural immunity, three grades of cervical lesions, and three cervical cancer stages), screening and treatment, and vaccination. The model simulates type-specific HPV transmission through sexual activity (using four different risk groups and sexual mixing) and type-specific natural history of cervical cancer, from persistent HPV infection to cervical cancer. The model assumes that HPV vaccines are prophylactic and therefore have no therapeutic effect and do not alter the natural history of HPV infection and related diseases.^22^

We simulated the impact of different HPV vaccination scenarios on four LMICs (ie, India, Nigeria, Uganda, and Viet Nam); these countries represent different profiles of sexual activity and HPV epidemiology.^20^

### Procedures

We used the HPV-ADVISE LMIC model for the projections and implemented the model in C++ (version 11). We parameterised and calibrated the model to the four countries separately using highly stratified country-specific sexual behaviour (eg, age-specific rates of first sexual experience, and lifetime number of partners) and epidemiological data (eg, age-specific HPV prevalence and cervical cancer incidence) taken from published articles and international population-based datasets (appendix 1 pp 6–46).

To represent uncertainty in sexual behaviour and HPV epidemiology within each country, we identified 20 parameter sets that simultaneously fit sexual activity and epidemiological data for each country (appendix 1 pp 47–51). We did the analysis and report our results according to HPV-FRAME,^23^ a consensus-based framework for modelled evaluations of HPV prevention and cervical cancer control (appendix 2 pp 3–4).

To estimate the population-level effectiveness and efficiency of one-dose vaccination, we compared one-dose routine vaccination of girls aged 9 years (combined with one-dose multi-age cohort vaccination of girls aged 10–14 years) with two-dose routine vaccination of girls aged 9 years (combined with two-dose multi-age cohort vaccination of girls aged 10–14 years). Multi-age cohort vaccination of girls aged 10–14 years was set to occur within the first year of the programme with the same vaccination coverage as routine vaccination. The base-case scenario assumes that one-dose or two-dose HPV vaccination is introduced in 2023 with 80% vaccination coverage, which was the median coverage in LMICs for the first dose in 2019.^3^ We also examined the potential effectiveness of a mitigation scenario: switching from one-dose to two-dose routine vaccination (combined with a one-dose catch-up vaccination of girls aged 10–14 years who would have received only one dose during the 5 years of one-dose routine vaccination), should ongoing studies show evidence that one-dose protection starts waning substantially within the next 5 years (by 2028, 18 years of one-dose efficacy^12^, ^13^ and 22 years of immunogenicity data will be available).^14^, ^15^

We also did sensitivity analyses to account for the different LMIC contexts. First, we examined the potential effectiveness of one-dose vaccination assuming a lower vaccination coverage of 40% (representing the lower quartile of coverage estimates in LMICs) or a higher coverage of 90% (representing the cervical cancer elimination target).^3^, ^24^ Second, we examined the potential effectiveness of switching from two-dose to one-dose routine vaccination, 5 years into the programme (compared with our base-case scenario where HPV vaccination is introduced starting with one or two doses).

For countries that delayed the introduction of two-dose routine vaccination and would like to accelerate the effectiveness of HPV vaccination, we examined a scenario of adding one-dose or two-dose multi-age cohort vaccination to two-dose routine vaccination to catch up unvaccinated girls before they age out of the school-based HPV vaccination window of 10–14 years. We assumed multi-age cohort vaccination coverage of 80% and 50% (due to potential drop in school attendance for older girls).

All vaccination scenarios for the main and secondary analyses are described in detail in appendix 2 (pp 5–6). Of note, given the focus on the effectiveness of HPV vaccination, our scenarios include the status quo for cervical cancer screening in each country.

For all scenarios, we used the nonavalent vaccine and assumed that two doses provide 100% efficacy against HPV types (6, 11, 16, 18, 31, 33, 45, 52, and 58) and a lifetime duration of protection.^25^, ^26^ On the basis of the India IARC trial, the CVT trial, and the KEN-SHE randomised controlled trial suggesting similar sustained protection for one and two doses, we examined a non-inferior one-dose scenario, in which one-dose vaccine efficacy and duration of protection are the same as for two doses.^12^, ^13^, ^14^, ^15^, ^16^, ^17^, ^18^ We also examined pessimistic scenarios of lower vaccine efficacy (85%) or average duration of protection (20 or 30 years). The pessimistic 85% vaccine efficacy represents the lower bound of the one-dose vaccine efficacy 95% CI in the India IARC trial (95% CI 85–100%).^12^ The 20 and 30 years of average protection were chosen as pessimistic scenarios given that there was no evidence of waning in vaccine protection after one dose through more than 10 years of follow-up in the Indian and CVT studies.^12^, ^13^, ^14^, ^15^ Duration of protection was modelled using a normal distribution with an average duration of protection of 20 or 30 years, and a 5-year standard deviation. The normal distribution was chosen as it allows reproduction of a stable vaccine efficacy for a set number of years before rapid waning occurs (appendix 2 p 19).

### Outcomes

We had three main outcomes. To examine the population-level effectiveness of the HPV vaccination scenarios, we used the relative reduction in cervical cancer incidence over time versus no vaccination (percent change in cervical cancer incidence) and the cumulative number of cervical cancers averted over 100 years. To examine the efficiency of the vaccination scenarios, we estimated the number of vaccine doses needed to prevent one cervical cancer by dividing the cumulative number of doses given in the population by the cumulative number of cervical cancers averted over 100 years. In the main analysis, the number of doses needed to prevent one cervical cancer was calculated using no vaccination as the comparator for one-dose and two-dose vaccination scenarios and, in the secondary analysis, the number of doses needed to prevent one cervical cancer for multi-age cohort vaccination were calculated using routine-only vaccination as the comparator. Cervical cancers and number of doses were calculated using age-specific and country-specific population projections from 2023 to 2123 from the UN World Population Prospects (appendix 2 p 28).^27^ For all outcomes, we present the mean of the 20 parameter sets that best fit country-specific sexual activity, HPV epidemiology, and Globocan 2020 cervical cancer incidence (appendix 1 pp 47–51). We also present the 80% uncertainty interval (10th and 90th percentiles), obtained from 400 simulations for each scenario (20 parameter sets and 20 simulations per parameter set). Given that cervical cancer can take several decades to develop after infection, time horizon was set to 100 years to capture the full effectiveness of the different HPV vaccination strategies. A 100-year time horizon is also consistent with the cervical cancer elimination goal, as this timeframe is needed to capture the full dynamics that would lead to the elimination of cervical cancer in current and subsequent generations.^28^

### Role of the funding source

The funders of the study had no role in study design, data collection, data analysis, data interpretation, or writing of the report.

## Results

**Figure 1:**
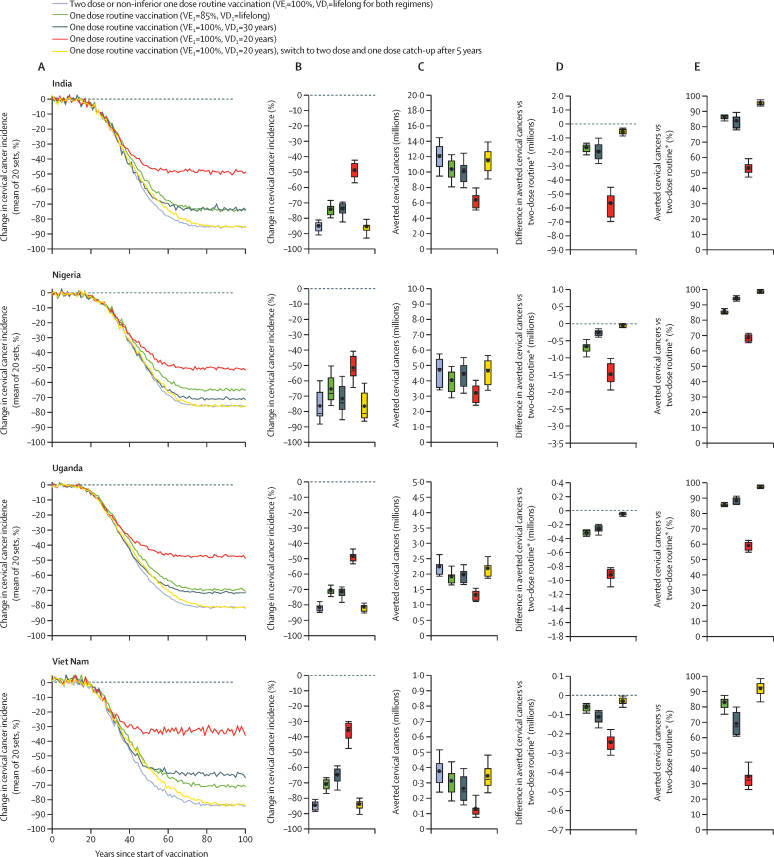
Projected population-level effectiveness of one-dose and two-dose routine vaccination of girls aged 9 years (with multi-age cohort vaccination of girls aged 10–14 years) assuming 80% vaccination coverage (A) Change in cervical cancer incidence over time since start of vaccination (*vs* no vaccination). (B) Change in cervical cancer incidence after 100 years (*vs* no vaccination). (C) Averted cervical cancers over 100 years after start of vaccination (*vs* no vaccination), in millions. (D) Difference in averted cervical cancers (*vs* two-dose vaccination), in millions. (E) Percentage of averted cervical cancers (*vs* two-dose vaccination). Error bars are 90th and 10th percentiles of the 20 parameter sets, boxes are the 25th and 75th percentiles, horizontal central lines within boxes are medians, and circles are means. Routine vaccination is combined with multi-age cohort vaccination of girls aged 10–14 years (within the first year of vaccination, with the same number of doses and coverage as routine). Vaccination was assumed to start in 2023. Vaccination coverage for routine and multi-age cohort vaccination was 80%. In all scenarios, VE_2_ is 100%, and VD_2_ is lifelong. Of note, uncertainty intervals should not be interpreted as confidence intervals from a statistical point of view. Uncertainty intervals reflect uncertainty in model parameters and variability in human papillomavirus epidemiology within a country. To compare the results between vaccination strategies, the uncertainty intervals in figures D and E should be used. VDi=vaccine duration of protection of dose *i*. VEi=vaccine efficacy of dose *i*.

In our main analysis, the model projects that one-dose routine vaccination of girls aged 9 years with 80% vaccination coverage would produce important reductions in cervical cancer incidence, if duration of protection is greater than 20–30 years (figure 1; appendix 2 pp 7–18). Two-dose routine vaccination of girls aged 9 years (with vaccination of a multi-age cohort of 10–14 years) or a similar strategy but with a non-inferior one-dose vaccine, would reduce cervical cancer incidence by 76% to 85% after 100 years (figure 1A, 1B; appendix 2 pp 7–18), and would avert 12·0 million cervical cancers in India, 4·7 million in Nigeria, 2·3 million in Uganda, and 0·4 million in Viet Nam over 100 years (figure 1C; appendix 2 pp 7–18). Under pessimistic assumptions of one-dose with 85% efficacy or a 30-year duration of protection, one-dose routine vaccination of girls aged 9 years would reduce cervical cancer incidence by 65% to 74% after 100 years depending on the country (figure 1A, 1B; appendix 2 pp 7–18), averting 69% to 94% (three of the four LMICs ≥84%) of the cervical cancers that would be averted with two-dose routine vaccination over 100 years (figure 1E; appendix 2 pp 7–18). However, under the most pessimistic assumption of one-dose with a 20-year duration of protection, the model projects that one-dose vaccination would avert substantially fewer cervical cancers in the four LMICs (36% to 52% reduction in cervical cancer incidence, averting 35% to 69% [three of four LMICs ≥53%] of the cervical cancers averted with two-dose vaccination, depending on the country; figure 1A, 1B, 1E; appendix 2 pp 7–18). Should studies start showing evidence that one-dose protection is waning substantially within 5 years (ie, by 2028), a potential switch to two-dose routine vaccination of girls aged 9 years, combined with one-dose catch-up vaccination of girls aged 10–14 years (irrespective of their vaccination status), could mitigate the effects of the short duration of protection and produce similar reductions in cervical cancer incidence and cervical cancers averted as a two-dose vaccination introduced in 2023 (77% to 85% reduction in cervical cancer incidence, averting 92% to 99% of the cervical cancers averted with two-dose vaccination, depending on the country; figure 1A, 1B, 1E; appendix 2 pp 7–18).

**Figure 2:**
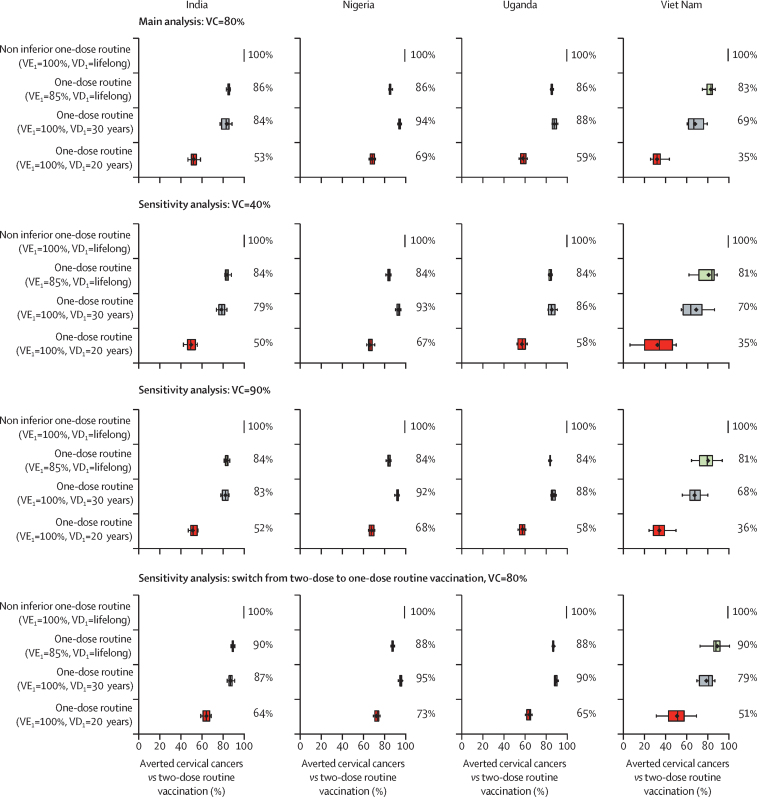
Projected percentage of averted cervical cancers with one-dose versus two-dose routine vaccination of girls aged 9 years, for different vaccination programme assumptions Error bars are 90th and 10th percentiles of the 20 parameter sets, boxes are the 25th and 75th percentiles, vertical central lines in each box are medians, and diamonds are means. Routine vaccination is combined with multi-age cohort vaccination of girls aged 10–14 years (within the first year of vaccination, with the same number of doses and coverage as routine). Vaccination is assumed to start in 2023. In all scenarios, VE_2_ is 100%, and VD_2_ is lifelong. VC=vaccination coverage. VD_i_=vaccine duration of protection of dose *i*. VE_i_=vaccine efficacy of dose *i*. Uncertainty intervals reflect uncertainty in model parameters and variability in HPV epidemiology within a country.

In the sensitivity analysis, the above conclusions were similar when assuming 40% or 90% vaccination coverage, or when assuming a switch from two-dose to one-dose routine vaccination of girls aged 9 years, 5 years after the start of the vaccination programme (figure 2; appendix 2 pp 7–18, 20–22). However, the overall reduction in cervical cancer incidence depends on vaccination coverage assumptions. Of note, for the same number of vaccine doses per year in a country, the population-level effectiveness of vaccination is projected to be higher by vaccinating 80% of girls with one dose (even under pessimistic one-dose assumptions), than vaccinating 40% of girls with two doses (assuming 100% lifelong protection of two doses; appendix 2 p 23).

**Figure 3:**
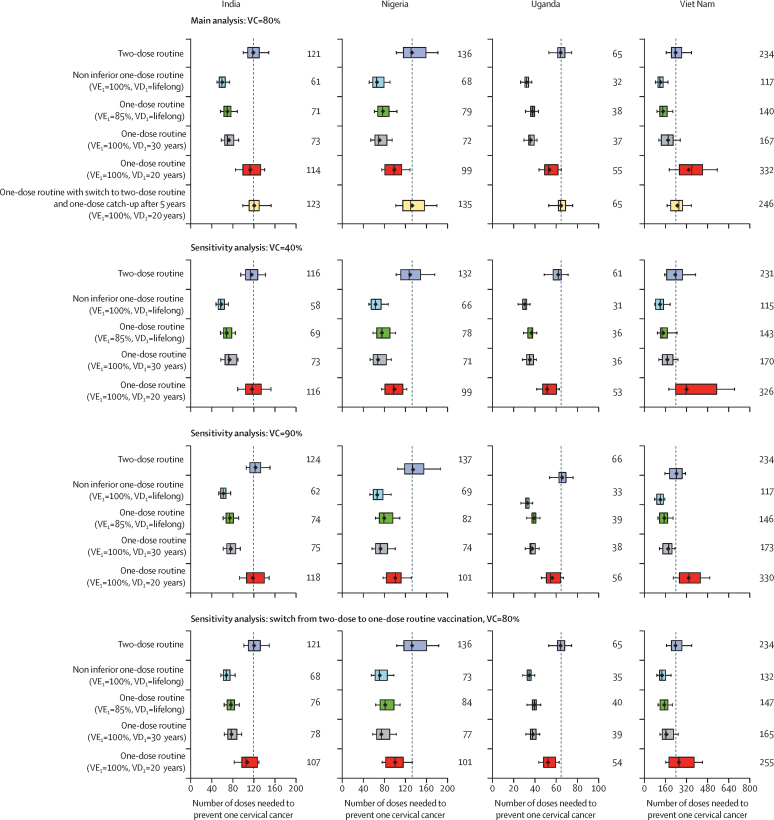
Projected number of doses needed to prevent one cervical cancer with one-dose or two-dose routine vaccination of girls aged 9 years (*vs* no vaccination), for different vaccination programme assumptions Error bars are 90th and 10th percentiles of the 20 parameter sets, boxes are the 25th and 75th percentiles, vertical central lines in boxes are medians, and diamonds are means. Routine vaccination is combined with multi-age cohort vaccination of girls aged 10–14 years (within the first year of vaccination, with the same number of doses and coverage as routine). Vaccination is assumed to start in 2023. In all scenarios, VE_2_ is 100%, and VD_2_ is lifelong. VC=vaccination coverage. VD_i_=vaccine duration of protection of dose *i*. VE_i_=vaccine efficacy of dose *i*. Of note, uncertainty intervals should not be interpreted as confidence intervals from a statistical point of view. Uncertainty intervals reflect uncertainty in model parameters and variability in HPV epidemiology within a country.

In terms of efficiency, the model projects that one-dose routine vaccination of girls aged 9 years (with a multi-age cohort vaccination of girls aged 10–14 years) would require substantially fewer doses to prevent one cervical cancer than two-dose vaccination, if duration of protection is at least 20–30 years (figure 3; appendix 2 pp 7–18). The number of doses needed to prevent one cervical cancer for two-dose routine vaccination, compared with no vaccination, were 121 in India, 136 in Nigeria, 65 in Uganda, and 234 in Viet Nam. The number of doses needed to prevent one cervical cancer with a non-inferior one-dose vaccine would be half these values (61 in India, 68 in Nigeria, 32 in Uganda, and 117 in Viet Nam). Under pessimistic assumptions of one-dose 85% efficacy or a 30-year duration of protection, one-dose routine vaccination of girls aged 9 years would be more efficient than two-dose routine vaccination in the four LMICs (number of doses needed to prevent one cervical cancer compared with no vaccination were 71–73 in India, 72–79 in Nigeria, 37–38 in Uganda, and 140–167 in Viet Nam). Under the most pessimistic assumption of a 20-year duration of protection, one-dose vaccination produced a similar number of doses needed to prevent one cervical cancer as two-dose vaccination with 80% uncertainty intervals overlapping (number of doses needed to prevent one cervical cancer compared with no vaccination were 114 in India, 99 in Nigeria, 55 in Uganda, and 332 in Viet Nam; appendix 2 pp 7–18). Varying vaccination coverage had little effect on the estimated number of doses needed to prevent one cervical cancer for one-dose or two-dose vaccination (figure 3; appendix 2 pp 7–18). Finally, switching from a one-dose to two-dose vaccination after 5 years resulted in similar estimates for the number of doses needed to prevent one cervical cancer as starting with two-dose vaccination in 2023 (figure 3; appendix 2 pp 7–18).

In our secondary analysis, the model projects that adding multi-age cohort vaccination to routine vaccination substantially accelerates the reduction in cervical cancer incidence and therefore produces a greater number of cumulative cervical cancers averted but does not affect cervical cancer incidence after 100 years. When adding two-dose multi-age cohort vaccination of girls aged 10–14 years with 80% vaccination coverage (or one-dose multi-age cohort vaccination with non-inferior vaccine efficacy and duration) to two-dose routine vaccination, the model projects an additional 5% to 14% of cervical cancers would be averted over 100 years (appendix 2 pp 7–18, 24). One-dose multi-age cohort vaccination of girls aged 10–14 years with 50% or 80% vaccination coverage would produce similar numbers of additional cervical cancers averted as two-dose multi-age cohort vaccination (assuming the same vaccination coverage), even under pessimistic assumptions of 85% one-dose vaccine efficacy, or 20-year or 30-year duration of protection (an additional 2% to 12% of cervical cancers averted, compared with two-dose routine vaccination; appendix 2 pp 7–18, 24, 25). In terms of efficiency, the model projects that adding one-dose multi-age cohort vaccination to two-dose routine vaccination would result in needing fewer doses to avert one cervical cancer than adding two-dose multi-age cohort vaccination (appendix 2 pp 7–18, 26).

## Discussion

Our results show that one-dose HPV routine vaccination of girls in LMICs would prevent 69% to 94% of the cervical cancers averted by two-dose vaccination over 100 years, under pessimistic assumptions of one-dose protection (85% vaccine efficacy or 30-year duration of protection). Furthermore, one-dose routine vaccination would be a more efficient use of available doses compared with two-dose vaccination. However, if the duration of protection for one dose is 20 years, one-dose vaccination would avert substantially fewer cervical cancers than two-dose vaccination (35% to 69% of the cervical cancers averted by two-dose vaccination, ≥53% in three of four LMICs). If this scenario were to occur, a switch after 5 years to two-dose vaccination could mitigate this loss in cervical cancer prevention. These conclusions were similar when assuming 40% or 90% vaccination coverage. Finally, adding one-dose or two-dose multi-age cohort vaccination of girls aged 10–14 years to routine vaccination would accelerate cervical cancer incidence reductions and avert an important additional number of cervical cancers over 100 years, even under pessimistic assumptions of one-dose protection.

Three recent modelling studies have examined the potential population-level effectiveness of one-dose HPV vaccination in LMICs.^29^, ^30^, ^31^ First, a comparative modelling study^29^ that we did with colleagues from Harvard University, found that early implementation of one-dose routine vaccination of girls in Uganda would lead to greater health benefits than waiting 5 years until more information on vaccine efficacy is available from ongoing trials. The conclusion was robust across a range of revaccination mitigation scenarios. Second, a modelling study^30^ projecting the potential effectiveness of HPV vaccination in China showed that one-dose vaccination of girls aged 14 years could avert 81–98% of the cervical cancers that would be averted with two-dose vaccination over 100 years (assuming 85% vaccine efficacy). Finally, a modelling study^31^ from IARC projected that one-dose vaccination of girls aged 10 years in India could reduce the lifetime risk of cervical cancer by 71–78% (depending on vaccine efficacy assumptions) and would be an efficient use of HPV vaccine doses. The results of the IARC modelling study are consistent with those for India presented in our study, when assuming one-dose vaccine efficacy is 85–100% and 80% vaccination coverage. However, the studies from China and India did not explore the effect of more pessimistic duration of vaccination protection scenarios. We have expanded on these studies by examining the effectiveness of one-dose HPV vaccination in four LMICs with differences in sexual behaviours and cervical cancer burden (India, Nigeria, Uganda, and Viet Nam) and by exploring a range of vaccination programme assumptions.

Our modelling study suggests that the duration of vaccine protection is the key determinant of one-dose versus two-dose population-level effectivenesss. One-dose protection must remain high for at least 20 years for girls and women to be protected during their peak ages of sexual activity. We project a substantial rebound in HPV incidence if duration of protection is 20 years on average but this effect will be much smaller if the duration of protection is 30 years (appendix 2 p 27). There were some differences by country, which were mainly explained by variability in sexual activity, HPV prevalence and cervical cancer incidence. Of note, the larger uncertainty intervals for the scenarios with a one-dose duration of protection of 30 years suggest that some countries (or regions) might require a slightly longer average duration of protection for one-dose vaccination to produce more than 80% of the effectiveness of two-dose vaccination; for example, countries where sexual activity starts or declines at an older age, in which female sex workers are older, or where men are an important population for infection transmission between younger and older women. However, a rebound in HPV incidence would be in older women who have fewer remaining life-years to develop cervical cancer (appendix 2 p 27), thus attenuating its effect on cervical cancer incidence. Furthermore, the average duration of one-dose protection is unlikely to be less than 20 years given that multiple studies^12^, ^13^, ^14^, ^15^ have shown a sustained protection and stable antibody titres for at least 10 years after one-dose vaccination. If the average duration of protection was less than 20 years, we would probably already have observed the first signs of declines in efficacy or antibody titres in trials (appendix 2 p 19). Therefore, in our shortest duration of protection scenario, we used a normal distribution of 20 years, where vaccine efficacy declines abruptly after 15 years, instead of a constant waning function (appendix 2 p 19). Given the importance of maintaining the duration of protection beyond 20 years, it is crucial to continue the long-term follow-up of the current trials addressing single-dose vaccination efficacy and to monitor vaccinated girls to detect any early signs of decreasing protection. If the duration of protection of one-dose vaccination is shown to wane within the next 5 years (ie, by 2028; at which time more than 15 years of follow-up will be available), implementing a mitigation strategy, such as switching to two-dose routine vaccination, could mitigate the potential losses in cervical cancer prevention. Other mitigation strategies could be explored, such as booster doses for women depending on the duration of one-dose protection and the vaccination coverage that could be achieved in these age groups. Countries could also decide to introduce extended schedules with a first dose at age 9 years and a potential second dose 5 years later at age 14 years, by which time more data on one-dose efficacy will be available.^20^, ^21^ Countries could then reassess whether it is necessary to give the second dose. However, changing a vaccination programme or implementing a mitigation strategy could cause logistical and communication challenges.

Our model projections have important policy implications for LMICs. One-dose vaccination could alleviate the programmatic and financial challenges related to two-dose schedules and thereby increase the speed of vaccine introduction, access, and coverage. One-dose HPV vaccination would be easier to implement, would be less costly, and would allow more programmatic flexibility (eg, multi-year multi-age campaigns) than two-dose vaccination. Introduction of one-dose vaccination could also allow saved doses to be redirected to vaccinate more cohorts of girls and women and boys and men to optimise vaccination impact and reduce inequities with HICs. For example, we have shown that one-dose multi-age cohort vaccination could be an effective and efficient use of available resources in countries that have yet to introduce HPV vaccination (or that have seen reductions in vaccination coverage due to the disruptions from the COVID-19 pandemic), where many girls are ageing out of the 9–14 years vaccination window. Partially based on the analyses presented in this study, WHO SAGE announced that one-dose or two-dose schedules could be considered for girls and young women aged 9–20 years.^19^

Our study has several strengths. First, we used HPV-ADVISE LMIC, which was calibrated with country-specific behavioural and epidemiological data to four LMICs. We chose two Asian and two African countries to represent different country profiles in terms of sexual activity and HPV epidemiology. Second, in past studies,^28^, ^32^ we have shown that the HPV-ADVISE model produces consistent results with other models for different policy questions. Third, our projections are based on 400 runs obtained from the best fitting 20 parameter sets that simultaneously fit country-specific data and capture uncertainty in sexual behaviour, HPV transmission, and natural history of HPV-related diseases. Finally, our model results are consistent across the four LMICs, despite the important differences in sexual behaviour and cervical cancer burden and are consistent for our different vaccination programme assumptions. Therefore, our conclusions are probably generalisable to a large number of LMICs where sexual behaviour indicators and HPV-related burden are within the range of our four LMIC profiles. However, our conclusions should not be generalised to HICs, as many HICs have had a high vaccination coverage for girls for more than a decade, have gender-neutral vaccination, high rates of cervical screening, and different sexual behaviours.

Our study also has a number of limitations, which must be considered when using and interpreting our results. First, we did not model all possible scenarios of vaccine efficacy, duration of protection, vaccination coverage, and vaccination target. However, we examined a wide range of vaccine assumptions. We examined a non-inferior scenario and pessimistic scenarios of vaccine efficacy and duration, which reflect the bottom 95% CI of the estimated one-dose vaccine efficacy in the India study (94%, 95% CI 84–99) and an average duration of 20 years.^12^, ^13^ We also assumed vaccination coverages of 40%, 80%, and 90%, which reflects the lower quartile and the median of the vaccination coverage with the first dose in LMICs in 2019, and the vaccination coverage target set by the WHO global strategy for cervical cancer elimination.^3^, ^24^ We did not model HPV vaccination beyond the primary target of girls aged 9–14 years, as the long-term effectiveness of one-dose or two-dose vaccination is mainly attributable to the routine strategy. Moreover, looking at vaccinating older girls and women is another policy question and demands examining many different scenarios to evaluate up to what age girls and women should be vaccinated, and whether it would be more efficient to vaccinate other groups, such as boys. Second, although we include uncertainty in sexual behaviour and natural history in our model projections, data on the number of new partners among older men and women and the rate of progression of HPV infection to cervical cancer in women infected after age 35 years remain sparse or incomplete. If the rates of partner acquisition or progression to cervical cancer are higher than modelled, then we could be underestimating the effect of a short duration of protection. Conversely, if these rates are lower than modelled then we might be overestimating the effect of duration of protection. Third, our model does not incorporate HIV. If one-dose efficacy is lower among individuals with HIV, our projections could overestimate the effectiveness of one-dose vaccination in countries with a high prevalence of HIV. Fourth, model projections are with the nonavalent vaccine. In the short term, LMICs might use the bivalent or quadrivalent vaccines. In a previous analysis, we have shown that the population-level effectiveness and efficiency are similar for the bivalent, quadrivalent, and nonavalent vaccines, particularly if there is cross-protection.^20^ Although the projected reductions in cervical cancer could be slightly lower for the bivalent and quadrivalent than the nonavalent vaccine, the main conclusions regarding one-dose vaccination would probably be similar for the different vaccines. Finally, substantial behavioural changes and technological development are anticipated over the 100-year time horizon of our analysis, which will affect cervical cancer incidence.

In conclusion, if the one-dose vaccine's duration of protection is greater than 20–30 years (depending on the LMIC), one-dose routine vaccination could avert most of the cervical cancers averted with two-dose vaccination, while being more efficient, easier to implement, and less costly. One-dose multi-age cohort vaccination could offer the opportunity to catch up girls before they age out of the 9–14 years vaccination window and could therefore mitigate the effect of delays in vaccination introduction or lower coverage during the COVID-19 pandemic. Finally, the doses saved through one-dose vaccination could provide the opportunity for LMICs to extend vaccination programmes to boys and older cohorts to maximise the population-level effectiveness of vaccination and reduce inequalities with HICs.

## Data sharing

No individual participant-level data were used in this study. Descriptions of the model structure, the parameters included in the model, and the empirical data used for calibration and validation are available in appendix 1 (pp 1–56).

## Declaration of interests

MB, MD, and MJ are members of the Single-Dose HPV Vaccine Evaluation Consortium, which is funded by the Bill & Melinda Gates Foundation. M-CB acknowledges funding from the MRC Centre for Global Infectious Disease Analysis (reference MR/R015600/1), jointly funded by the UK Medical Research Council and the UK Foreign, Commonwealth & Development Office, under the MRC/FCDO Concordat agreement and is also part of the EDCTP2 programme supported by the EU. All other authors declare no competing interests.
