# Supplementary material for: Potential population-level effectiveness of one-dose HPV vaccination in low-income and middle-income countries: a mathematical modelling analysis
Source: Lancet Public Health. 2023 Sep 28;8(10):e788–99. doi: 10.1016/S2468-2667(23)00180-9 (PMC10557953; doi:10.1016/S2468-2667(23)00180-9)
Supplement: Supplementary appendix 2 [file mmc2.pdf]

THE LANCET
Public Health

Supplementary appendix 2

This appendix formed part of the original submission and has been peer reviewed. We post it as supplied by the authors.

Supplement to: Bénard É, Drolet M, Laprise J-F, et al. Potential population-level effectiveness of one-dose HPV vaccination in low-income and middle-income countries: a mathematical modelling analysis. *Lancet Public Health* 2023; **8:** e788–99.

Supplementary appendix

Supplement to: Bénard É, Drolet M, Laprise J-F, Gingras G, Jit M, Boily MC, Bloem P, Brisson M. Potential population-level effectiveness of one-dose HPV vaccination in low-income and middle-income countries: a mathematical modeling analysis.

Supplementary Appendix

Table S1: HPV-FRAME....................3

Table S2: Description of the HPV vaccination scenarios examined....................5

Table S3: Change in cervical cancer incidence at equilibrium, averted cases, and number of doses needed to prevent one cancer (NNV) in India, Vietnam, Uganda and Nigeria....................7

Figure S1: Waning of vaccine protection. 19

Figure S2: Projected population-level impact of one- and two-dose routine vaccination of 9-year-old girls (with MAC vaccination of 10-14-year-old girls) assuming 40% vaccination coverage. 20

Figure S3: Projected population-level impact of one- and two-dose routine vaccination of 9-year-old girls (with MAC vaccination of 10-14-year-old girls) assuming 90% vaccination coverage. 21

Figure S4: Projected population-level impact of switching from two- to one-dose routine vaccination of 9-year-old girls after 5 years, assuming 80% vaccination coverage. 22

Figure S5: Projected population-level impact of one- and two-dose routine vaccination of 9-year-old girls (with MAC vaccination of 10-14-year-old girls) assuming 80% vaccination coverage for one-dose scenarios and 40% vaccination coverage for two-dose scenario. 23

Figure S6: Projected population-level impact of one- and two-dose MAC vaccination of 10-14-year-old girls (with two-dose routine vaccination of 9-year-old girls) assuming 80% MAC vaccination coverage. 24

Figure S7: Projected population-level impact of one- and two-dose MAC vaccination of 10-14-year-old girls (with two-dose routine vaccination of 9-year-old girls) assuming 50% MAC vaccination coverage. 25

Figure S8: Number of doses needed to prevent one cervical cancer (NNV) through one- and two-dose MAC vaccination. 26

Figure S9: HPV-16 infection in women over time, mean age at infection, and mean age at cervical cancer. 27

Estimation of country-specific population size between 2100-2123....................28

References 29

Table S1: HPV-FRAME[1]

A. Inputs	Reported by age? (Y/N)	Reported by sex?	Comments
**Core reporting standard**			
Target population for intervention	Y	Y	Vaccination of girls aged 9-14 years (see section Methods of the article).
Sexual behavior	Y	Y	Sexual behavior inputs used (number of lifetime partners, number of partners in the last year, partnership formation, etc) are described in the appendix 1, section 2.2.
Cohort examined for evaluation/ time horizon	Y (multiple cohorts)	Y (multiple cohorts)	100-year time horizon from start of vaccination (2023-2123). Intervention is given to cohorts, but we examine the outcome in the population.
Quality of life assumptions	Not applicable	Not applicable	This study focuses on the impact of vaccination on health outcomes only (cervical cancer).
Calibration	Y	Y	HPV-ADVISE LMIC was calibrated with country-specific behavioral and epidemiological data (see appendix 1, section 2).
Validation (where possible)	Y	Y	HPV-ADVISE LMIC has been validated and previously used to model various HPV vaccination and cervical cancer screening strategies (see appendix 1, section 2.4 for details on validation). We have also performed comparative model validation with other independent models for different policy questions and projections were consistent between models.[2,3]
Costs	Not applicable	Not applicable	This study focuses on the impact of vaccination on health outcomes only (cervical cancer).
**Reporting standards for models of vaccination in adolescent individuals**			
Vaccine coverage	Y	Y	Intervention is for girls only. See section Methods of the article for specific coverage.
Vaccine efficacy	Y	Y	See section Methods of the article for specific efficacy.
Vaccine cross-protection	Not applicable	Not applicable	The study focuses on the nonavalent vaccine, which includes oncogenic types HPV16, 18, 31, 33, 45, 52 and 58.
Duration vaccine protection and waning	Y	Y	See section Methods of the article for assumptions on duration of protection.
Vaccine and delivery costs	Not applicable	Not applicable	This study focuses on the impact of vaccination on health outcomes only (cervical cancer).
Pre-vaccination disease burden	Y	Y	Pre-vaccination epidemiological data were used for calibration (see appendix 1, section 2.3).
Duration of natural immunity	Y	Y	See appendix 1, section 2.2.

A. Inputs	Reported by age? (Y/N)	Reported by sex?	Comments
**Reporting standards for evaluations assessing alternative vaccine types or reduced-dose schedules**			
Timing between doses	Y	Not applicable	Not applicable for one-dose schedules. Six months between doses for two-dose schedule. Girls-only vaccination.
**Reporting standards for models of HPV prevention in LMIC**			
HIV prevalence rates, if endemic in country	N	N	We did not account for HIV prevalence (see section Discussion of the article).
Description of any opportunistic or pilot/demonstration screening projects ongoing	N	N	See appendix 1, section 2.2 for screening assumptions.

B. Outputs	Reported by age? (Y/N)	Report by sex?	Comments
**Core reporting standards**			
Cancer incidence, mortality, life years, QALYs/DALYs (as appropriate)	Y	Y	We present the absolute number of cervical cancer cases averted, the number of doses needed to prevent one cancer (NNV) and the relative reduction in cervical cancer incidence (see section Methods and Results of the article).
HPV prevalence, pre-intervention	N	N	Pre-intervention prevalence is used for calibration and is reported in the appendix 1, section 2.3.
CIN2/3 detected	N	N	This outcome is not reported as the present study focuses on the impact of vaccination on cervical cancer.
Sensitivity analysis on key inputs	Y	Y	Sensitivity analyses were performed. See sections Methods and Results of the article.
Incremental cost-effectiveness ratios and costs saved	Not applicable	Not applicable	This study focuses on the impact of vaccination on health outcomes only (cervical cancer).
**Reporting standards for models of vaccination in adolescent individuals**			
Absolute reductions in HPV infections, and/or warts, post-vaccination	See comment	Y	Absolute reductions in HPV 16 incidence post-vaccination are presented in the appendix 2 figure S9 (p.27).
Absolute reductions in CIN2+ post-vaccination	N	N	Absolute reductions in CIN2+ are not presented since this study focuses on the impact of vaccination on cervical cancer.
Absolute reductions in invasive cancer (cervical and other HPV cancers, as relevant) post-vaccination	See comment	Y	We present the absolute number of cervical cancer cases averted and the relative reduction in cervical cancer incidence (see section Methods and Results of the article).

Table S2: Description of the HPV vaccination scenarios examined

Vaccination scenarios	Vaccine efficacy	Duration of protection	Vaccination coverage
**1. Main analysis**			
1.a Base case analysis of the main analysis			
**Comparator:** Two-dose routine vaccination of 9-year-old girls + two-dose multi-age cohort (MAC)* vaccination of 10-14-years old girls	100%	lifetime	80%
**One-dose scenarios:** One-dose routine vaccination of 9-year-old girls + one-dose MAC vaccination of 10-14-years old girls			
• Non-inferior one-dose vs two doses	100%	lifetime	80%
• Pessimistic one-dose efficacy	85%	lifetime	80%
• Pessimistic one-dose duration	100%	30 years	80%
• Pessimistic one-dose duration	100%	20 years	80%
**Mitigation strategy:** Switch to two-dose routine of 9-year-old girls (+ one-dose catch-up of 10-14-year-old girls) after 5 years of one-dose routine vaccination, assuming a 20-year duration of one-dose protection			
• One-dose routine vaccination for 5 years and<br>switch to two doses + one-dose catch-up of 10-14-year-old	100%<br>100%	20 years<br>lifetime	80%<br>80%
1.b Sensitivity analyses of the main analysis			
**Low coverage:** Two- or one-dose routine vaccination of 9-year-old girls + two- or one-dose MAC vaccination of 10-14-years old girls			
• Two-dose	100%	lifetime	40%
• Non-inferior one-dose vs two doses	100%	lifetime	40%
• Pessimistic one-dose efficacy	85%	lifetime	40%
• Pessimistic one-dose duration	100%	30 years	40%
• Pessimistic one-dose duration	100%	20 years	40%
**High coverage (elimination target):** Two-or one-dose routine vaccination of 9-year-old girls + two- or one-dose MAC vaccination of 10-14-years old girls			
• Two-dose	100%	lifetime	90%
• Non-inferior one-dose vs two doses	100%	lifetime	90%
• Pessimistic one-dose efficacy	85%	lifetime	90%
• Pessimistic one-dose duration	100%	30 years	90%
• Pessimistic one-dose duration	100%	20 years	90%
**Switch from two- to one-dose vaccination program:** One-dose routine vaccination of 9-year-old girls after five years of two-dose vaccination			
• Non-inferior one-dose vs two doses	100%	lifetime	80%
• Pessimistic one-dose efficacy	85%	lifetime	80%
• Pessimistic one-dose duration	100%	30 years	80%
• Pessimistic one-dose duration	100%	20 years	80%

Vaccination scenarios	Vaccine efficacy	Duration of protection	Vaccination coverage
**2. Secondary analysis**			
2.a Base case analysis of the secondary analysis			
**Comparator:** Two-dose routine vaccination of 9-year-old girls	100%	lifetime	80%
**Adding MAC vaccination:** Two-dose routine vaccination of 9-year-old girls + two- or one-dose MAC of 10-14-year-old girls			
• Two-dose MAC	100%	lifetime	80%
• One-dose MAC, non-inferior one-dose	100%	lifetime	80%
• One-dose MAC, pessimistic one-dose efficacy	85%	lifetime	80%
• One-dose MAC, pessimistic one-dose duration	100%	30 years	80%
• One-dose MAC, pessimistic one-dose duration	100%	20 years	80%
2.b Sensitivity analyses of the secondary analysis			
**Adding MAC vaccination- low coverage:** Two-dose routine vaccination of 9-year-old girls + Two- or one-dose MAC of 10-14-year-old girls			
• Two-dose MAC	100%	lifetime	50%
• One-dose MAC, non-inferior	100%	lifetime	50%
• One-dose MAC, pessimistic one-dose efficacy	85%	lifetime	50%
• One-dose MAC, pessimistic one-dose duration	100%	30 years	50%
• One-dose MAC, pessimistic one-dose duration	100%	20 years	50%

* Routine vaccination is combined with multi-age cohort (MAC) vaccination of 10-year-old girls (with the same number of doses).

Table S3: Change in cervical cancer incidence at equilibrium, averted cases, and number of doses needed to prevent one cancer (NNV) in India, Vietnam, Uganda and Nigeria

	Vaccine efficacy	Duration of protection	Vaccination coverage	Change in cervical cancer incidence after 100 years (%) Mean (80% UI+)	Averted cervical cancers (millions) Mean (80% UI)	Difference in averted cervical cancers vs ref (millions) Mean (80% UI)	Percentage of averted cervical cancers vs ref (%) Mean (80% UI)	Total number of doses (millions) Mean (80% UI)	NNV¥ Mean (80% UI)
**INDIA**									
**1. Main analysis**									
1.a Base case analysis of the main analysis									
**Comparator:** Two-dose routine vaccination of 9-year-old girls + two-dose MAC* vaccination of 10-14-years old girls	100%	lifetime	80%	-85.0% (-90.9% to -81.1%)	12.04 (9.46 to 14.45)	Ref	Ref	1,461	121 (101 to 149)
**One-dose scenarios:** One-dose routine vaccination of 9-year-old girls + one-dose MAC vaccination of 10-14-years old girls									
• Non-inferior one-dose vs two doses	100%	lifetime	80%	-85.0% (-90.9% to 81.1%)	12.04 (9.46 to 14.45)	0 (0 to 0)	100% (100% to 100%)	730	61 (50 to 74)
• Pessimistic one-dose efficacy	85%	lifetime	80%	-74.2% (-79.7% to -68.3%)	10.36 (8.109to 12.25)	-1.68 (-2.20 to -1.37)	86.1% (83.6% to 87.5%)	730	71 (57 to 89)
• Pessimistic one-dose duration	100%	30 years	80%	-73.8% (-82.4% to -69.4%)	10.06 (7.98 to 12.44)	-1.97 (-2.82 to -1.01)	83.6% (77.8% to 89.2%)	730	73 (59 to 91)
• Pessimistic one-dose duration	100%	20 years	80%	-48.8% (-56.9% to -42.2%)	6.40 (5.10 to 7.96)	-5.64 (-6.95 to -4.50)	53.1% (47.1% to 59.0%)	730	114 (86 to 141)
**Mitigation strategy:** One-dose routine vaccination for 5 years and switch to two doses + one-dose catch-up of 10-14-year-old	100%<br>100%	20 years<br>lifetime	80%<br>80%	-85.4% (-92.9% to -80.7%)	11.48 (9.12 to 13.90)	-0.56 (-0.85 to -0.29)	95.4% (93.4% to 97.4%)	1,414	123 (99 to 154)
1.b Sensitivity analyses of the main analysis									
**Low coverage:** Two- or one-dose routine vaccination of 9-year-old girls + two- or one-dose MAC vaccination of 10-14-years old girls									
• Two-dose	100%	lifetime	40%	-44.3% (-52.5% to -37.6%)	6.32 (4.94 to 7.41)	Ref	Ref	730	116 (95 to 142)

	Vaccine efficacy	Duration of protection	Vaccination coverage	Change in cervical cancer incidence after 100 years (%)<br>Mean (80% UI[+])	Averted cervical cancers (millions)<br>Mean (80% UI)	Difference in averted cervical cancers vs ref (millions)<br>Mean (80% UI)	Percentage of averted cervical cancers vs ref (%)<br>Mean (80% UI)	Total number of doses (millions)<br>Mean (80% UI)	NNV[¥]<br>Mean (80% UI)
• Non-inferior one-dose vs two doses	100%	lifetime	40%	-44.3%<br>(-52.5% to -37.6%)	6.32<br>(4.94 to 7.41)	0<br>(0 to 0)	100%<br>(100% to 100%)	365	58<br>(47 to 71)
• Pessimistic one-dose efficacy	85%	lifetime	40%	-37.9%<br>(-44.6% to -34.5%)	5.33<br>(4.20 to 6.44)	-0.99<br>(-1.29 to -0.70)	84.3%<br>(81.8% to 88.1%)	365	69<br>(56 to 84)
• Pessimistic one-dose duration	100%	30 years	40%	-37.0%<br>(-44.3% to -29.8%)	5.02<br>(3.88 to 6.17)	-1.30<br>(-1.79 to -0.95)	79.3%<br>(73.7% to 83.9%)	365	73<br>(57 to 89)
• Pessimistic one-dose duration	100%	20 years	40%	-24.0%<br>(-31.0% to -18.9%)	3.14<br>(2.33 to 4.05)	-3.18<br>(-3.86 to -2.53)	49.6%<br>(42.4% to 55.6%)	365	116<br>(89 to 152)
**High coverage (elimination target):**<br>Two- or ne-dose routine vaccination of 9-year-old girls<br>+ two- or one-dose MAC vaccination of 10-14-years old girls									
• Two-dose	100%	lifetime	90%	-93.0%<br>(-97.9% to -88.5%)	13.23<br>(10.32 to 15.74)	Ref	Ref	1,643	124<br>(104 to 149)
• Non-inferior one-dose vs two doses	100%	lifetime	90%	-93.0%<br>(-97.9% to -88.5%)	13.23<br>(10.32 to 15.74)	0<br>(0 to 0)	100%<br>(100% to 100%)	821	62<br>(52 to 75)
• Pessimistic one-dose efficacy	85%	lifetime	90%	-81.7%<br>(-89.1% to -77.3%)	11.11<br>(8.92 to 13.39)	-2.12<br>(-2.58 to -1.61)	83.9%<br>(81.2% to 86.5%)	821	74<br>(60 to 90)
• Pessimistic one-dose duration	100%	30 years	90%	-82.6%<br>(-92.0% to -77.0%)	10.92<br>(8.74 to 13.49)	-2.30<br>(-2.77 to -1.68)	82.5%<br>(78.0% to 85.9%)	821	75<br>(61 to 93)
• Pessimistic one-dose duration	100%	20 years	90%	-54.9%<br>(-61.5% to -47.5%)	6.95<br>(5.43 to 8.87)	-6.27<br>(-7.40 to -5.45)	52.4%<br>(47.1% to 56.3%)	821	118<br>(91 to 148)
**Switch from two- to one-dose vaccination program:**<br>One-dose routine vaccination of 9-year-old girls after five years of two-dose vaccination									
• Non-inferior one-dose vs two doses	100%	lifetime	80%	-85.0%<br>(-90.9% to -81.1%)	12.04<br>(9.46 to 14.45)	0<br>(0 to 0)	100%<br>(100% to 100%)	822	68<br>(57 to 84)
• Pessimistic one-dose efficacy	85%	lifetime	80%	-74.2%<br>(-80.2% to -69.2%)	10.77<br>(8.42 to 12.87)	-1.26<br>(-1.67 to -0.97)	89.5%<br>(87.5% to 91.1%)	822	76<br>(63 to 92)
• Pessimistic one-dose duration	100%	30 years	80%	-72.9%<br>(-83.3% to -68.2%)	10.53<br>(8.34 to 12.88)	-1.50<br>(-2.00 to -1.02)	87.4%<br>(83.9% to 91.1%)	822	78<br>(63 to 96)
• Pessimistic one-dose duration	100%	20 years	80%	-48.8%<br>(-53.8% to -44.1%)	7.71<br>(6.24 to 9.80)	-4.32<br>(-5.23 to -3.56)	64.0%<br>(58.7% to 68.6%)	822	107<br>(82 to 129)

	Vaccine efficacy	Duration of protection	Vaccination coverage	Change in cervical cancer incidence after 100 years (%)<br>Mean (80% UI[+])	Averted cervical cancers (millions)<br>Mean (80% UI)	Difference in averted cervical cancers vs ref (millions)<br>Mean (80% UI)	Percentage of averted cervical cancers vs ref (%)<br>Mean (80% UI)	Total number of doses (millions)<br>Mean (80% UI)	NNV[¥]<br>Mean (80% UI)
**2. Secondary analysis**									
2.a Base case analysis of the secondary analysis									
**Comparator:**<br>Two-dose routine vaccination of 9-year-old girls	100%	lifetime	80%	-85.4%<br>(-91.9% to -80.8%)	11.07<br>(8.62 to 13.22)	Ref	Ref	1,366	Ref
**Adding MAC vaccination:**<br>Two-dose routine vaccination of 9-year-old girls<br>+ two- or one-dose MAC of 10-14-year-old girls									
• Two-dose MAC	100%	lifetime	80%	-85.0%<br>(-90.9% to -81.1%)	12.04<br>(9.46 to 14.45)	0.96<br>(0.66 to 1.24)	108.7%<br>(106.5% to 110.4%)	1,461	98<br>(75 to 141)
• One-dose MAC, non-inferior one-dose	100%	lifetime	80%	-85.0%<br>(-90.9% to -81.1%)	12.04<br>(9.46 to 14.45)	0.96<br>(0.66 to 1.24)	108.7%<br>(106.5% to 110.4%)	1,413	49<br>(38 to 70)
• One-dose MAC, pessimistic one-dose efficacy	85%	lifetime	80%	-85.2%<br>(-92.1% to -80.7%)	11.83<br>(9.41 to 14.25)	0.76<br>(0.47 to 0.99)	106.9%<br>(104.8% to 108.3%)	1,413	62<br>(47 to 85)
• One-dose MAC, pessimistic one-dose duration	100%	30 years	80%	-85.3%<br>(-89.1% to -81.6%)	11.90<br>(9.57 to 14.27)	0.82<br>(0.41 to 1.06)	107.4%<br>(103.4% to 109.1%)	1,413	57<br>(43 to 92)
• One-dose MAC, pessimistic one-dose duration	100%	20 years	80%	-85.3%<br>(-91.2% to -81.6%)	11.64<br>(9.28 to 14.18)	0.57<br>(0.21 to 0.89)	105.1%<br>(102.1% to 107.4%)	1,413	83<br>(52 to 221)
2.b Sensitivity analyses of the secondary analysis									
**Adding MAC vaccination- low coverage:**<br>Two-dose routine vaccination of 9-year-old girls<br>+ two- or one-dose MAC of 10-14-year-old girls									
• Two-dose MAC	100%	lifetime	50%	-85.1%<br>(-91.7% to -81.9%)	11.66<br>(9.13 to 14.11)	0.59<br>(0.27 to 0.81)	105.2%<br>(102.8% to 106.7%)	1,425	101<br>(66 to 196)
• One-dose MAC, non-inferior	100%	lifetime	50%	-85.1%<br>(-91.7% to -81.9%)	11.66<br>(9.13 to 14.11)	0.59<br>(0.27 to 0.81)	105.2%<br>(102.8% to 106.7%)	1,396	51<br>(33 to 98)
• One-dose MAC, pessimistic one-dose efficacy	85%	lifetime	50%	-85.2%<br>(-91.5% to -81.9%)	11.57<br>(8.92 to 13.98)	0.50<br>(0.30 to 0.71)	104.6%<br>(102.7% to 106.1%)	1,396	59<br>(41 to 94)
• One-dose MAC, pessimistic one-dose duration	100%	30 years	50%	-85.2%<br>(-91.6% to -79.8%)	11.59<br>(9.20 to 13.98)	0.52<br>(0.28 to 0.69)	104.8%<br>(102.8% to 105.8%)	1,396	57<br>(39 to 81)
• One-dose MAC, pessimistic one-dose duration	100%	20 years	50%	-85.2%<br>(-91.0% to -81.3%)	11.43<br>(9.13 to 13.85)	0.36<br>(0.19 to 0.64)	103.3%<br>(101.8% to 105.0%)	1,396	82<br>(46 to 152)

	Vaccine efficacy	Duration of protection	Vaccination coverage	Change in cervical cancer incidence after 100 years (%)<br><br>Mean (80% UI[+])	Averted cervical cancers (millions)<br><br>Mean (80% UI)	Difference in averted cervical cancers vs ref (millions)<br><br>Mean (80% UI)	Percentage of averted cervical cancers vs ref (%)<br><br>Mean (80% UI)	Total number of doses (millions)<br><br>Mean (80% UI)	NNV[¥]<br><br>Mean (80% UI)
**VIETNAM**									
**1. Main analysis**									
1.a Base case analysis of the main analysis									
**Comparator:**<br>Two-dose routine vaccination of 9-year-old girls + two-dose MAC[*] vaccination of 10-14-years old girls	100%	lifetime	80%	-84.7%<br>(-88.8% to -80.8%)	0.38<br>(0.24 to 0.52)	Ref	Ref	88	234<br>(168 to 360)
**One-dose scenarios :**<br>One-dose routine vaccination of 9-year-old girls + one-dose MAC vaccination of 10-14-years old girls									
• Non-inferior one-dose vs two doses	100%	lifetime	80%	-84.7%<br>(-88.8% to -80.8%)	0.38<br>(0.24 to 0.52)	0<br>(0 to 0)	100%<br>(100% to 100%)	44	117<br>(84 to 180)
• Pessimistic one-dose efficacy	85%	lifetime	80%	-71.0%<br>(-77.0% to -66.7%)	0.31<br>(0.18 to 0.44)	-0.06<br>(-0.09 to -0.04)	82.8%<br>(75.2% to 87.4%)	44	140<br>(95 to 217)
• Pessimistic one-dose duration	100%	30 years	80%	-64.9%<br>(-74.8% to -59.0%)	0.26<br>(0.16 to 0.39)	-0.11<br>(-0.17 to -0.08)	68.9%<br>(60.9% to 79.8%)	44	167<br>(110 to 274)
• Pessimistic one-dose duration	100%	20 years	80%	-36.0%<br>(-47.7% to -30.2%)	0.13<br>(0.08 to 0.22)	-0.24<br>(-0.31 to -0.18)	34.6%<br>(26.1% to 44.0%)	44	332<br>(190 to 558)
**Mitigation strategy:**<br>One-dose routine vaccination for 5 years and switch to two doses + one-dose catch-up of 10-14-year-old	100%<br>100%	20 years<br>lifetime	80%<br>80%	-84.0%<br>(-90.6% to -80.0%)	0.35<br>(0.24 to 0.48)	-0.03<br>(-0.06 to 0.00)	91.8%<br>(83.2% to 98.3%)	85	246<br>(173 to 358)
1.b Sensitivity analyses of the main analysis									
**Low coverage :**<br>Two- or one-dose routine vaccination of 9-year-old girls + two- or one-dose MAC vaccination of 10-14-years old girls									
• Two-dose	100%	lifetime	40%	-45.0%<br>(-55.5% to -38.0%)	0.19<br>(0.11 to 0.28)	Ref	Ref	44	231<br>(155 to 390)

	Vaccine efficacy	Duration of protection	Vaccination coverage	Change in cervical cancer incidence after 100 years (%)<br>Mean (80% UI[+])	Averted cervical cancers (millions)<br>Mean (80% UI)	Difference in averted cervical cancers vs ref (millions)<br>Mean (80% UI)	Percentage of averted cervical cancers vs ref (%)<br>Mean (80% UI)	Total number of doses (millions)<br>Mean (80% UI)	NNV[¥]<br>Mean (80% UI)
• Non-inferior one-dose vs two doses	100%	lifetime	40%	-45.0% (-55.5% to -38.0%)	0.19 (0.11 to 0.28)	0 (0 to 0)	100% (100% to 100%)	22	115 (78 to 195)
• Pessimistic one-dose efficacy	85%	lifetime	40%	-36.2% (-43.3% to -30.8%)	0.15 (0.08 to 0.21)	-0.04 (-0.09 to -0.02)	80.9% (62.7% to 89.5%)	22	143 (98 to 248)
• Pessimistic one-dose duration	100%	30 years	40%	-33.1% (-43.8% to -23.0%)	0.13 (0.08 to 0.17)	-0.06 (-0.12 to -0.01)	70.0% (55.6% to 86.8%)	22	170 (110 to 256)
• Pessimistic one-dose duration	100%	20 years	40%	-18.7% (-32.2% to -10.5%)	0.07 (0.01 to 0.11)	-0.12 (-0.19 to -0.09)	34.7% (6.9% to 50.7%)	22	326 (186 to 687)
**High coverage (elimination target):**<br>Two- or ne-dose routine vaccination of 9-year-old girls<br>+ two- or one-dose MAC vaccination of 10-14-years old girls									
• Two-dose	100%	lifetime	90%	-93.5% (-98.7% to -90.0%)	0.42 (0.24 to 0.57)	Ref	Ref	99	234 (156 to 311)
• Non-inferior one-dose vs two doses	100%	lifetime	90%	-93.5% (-98.7% to -90.0%)	0.42 (0.24 to 0.57)	0 (0 to 0)	100% (100% to 100%)	50	117 (78 to 156)
• Pessimistic one-dose efficacy	85%	lifetime	90%	-80.7% (-87.8% to -75.8%)	0.34 (0.22 to 0.46)	-0.08 (-0.15 to -0.01)	81.4% (65.2% to 94.2%)	50	146 (103 to 217)
• Pessimistic one-dose duration	100%	30 years	90%	-71.8% (-78.8% to -66.2%)	0.29 (0.16 to 0.40)	-0.14 (-0.21 to -0.07)	68.0% (56.4% to 80.5%)	50	173 (116 to 232)
• Pessimistic one-dose duration	100%	20 years	90%	-39.8% (-47.5% to -34.1%)	0.15 (0.08 to 0.20)	-0.27 (-0.45 to -0.15)	36.3% (24.8% to 50.4%)	50	330 (220 to 497)
**Switch from two- to one-dose vaccination program:**<br>One-dose routine vaccination of 9-year-old girls after five years of two-dose vaccination									
• Non-inferior one-dose vs two doses	100%	lifetime	80%	-84.7% (-88.8% to -80.8%)	0.38 (0.24 to 0.52)	0 (0 to 0)	100% (100% to 100%)	49	132 (94 to 203)
• Pessimistic one-dose efficacy	85%	lifetime	80%	-73.2% (-79.7% to -66.7%)	0.34 (0.21 to 0.46)	-0.04 (-0.10 to 0.00)	89.9% (73.2% to 101.3%)	49	147 (104 to 212)
• Pessimistic one-dose duration	100%	30 years	80%	-64.7% (72.0% to -61.6-%)	0.30 (0.19 to 0.42)	-0.08 (-0.12 to -0.05)	79.2% (70.2% to 87.4%)	49	165 (118 to 257)
• Pessimistic one-dose duration	100%	20 years	80%	-36.2% (-51.3% to -24.2%)	0.19 (0.11 to 0.28)	-0.18 (-0.27 to -0.08)	51.3% (31.6% to 69.8%)	49	255 (163 to 441)

	Vaccine efficacy	Duration of protection	Vaccination coverage	Change in cervical cancer incidence after 100 years (%) Mean (80% UI[+])	Averted cervical cancers (millions) Mean (80% UI)	Difference in averted cervical cancers vs ref (millions) Mean (80% UI)	Percentage of averted cervical cancers vs ref (%) Mean (80% UI)	Total number of doses (millions) Mean (80% UI)	NNV[¥] Mean (80% UI)
**2. Secondary analysis**									
2.a Base case analysis of the secondary analysis									
**Comparator:** Two-dose routine vaccination of 9-year-old girls	100%	lifetime	80%	-83.9% (-89.2% to -78.1%)	0.33 (0.20 to 0.48)	Ref	Ref	82	Ref
**Adding MAC vaccination:** Two-dose routine vaccination of 9-year-old girls + two- or one-dose MAC of 10-14-year-old girls									
• Two-dose MAC	100%	lifetime	80%	-84.7% (-88.8% to -80.8%)	0.38 (0.24 to 0.52)	0.04 (0.02 to 0.06)	114.1% (106.8% to 120.0%)	88	127 (65 to 201)
• One-dose MAC, non-inferior one-dose	100%	lifetime	80%	-84.7% (-88.8% to -80.8%)	0.38 (0.24 to 0.52)	0.04 (0.02 to 0.06)	114.1% (106.8% to 120.0%)	85	63 (32 to 101)
• One-dose MAC, pessimistic one-dose efficacy	85%	lifetime	80%	-83.6% (-89.0% to -80.7%)	0.37 (0.23 to 0.51)	0.04 (0.02 to 0.06)	111.2% (105.1% to 117.8%)	85	77 (43 to 138)
• One-dose MAC, pessimistic one-dose duration	100%	30 years	80%	-83.8% (-87.5% to -80.8%)	0.37 (0.23 to 0.51)	0.04 (0.01 to 0.05)	112.1% (103.1% to 118.0%)	85	74 (42 to 176)
• One-dose MAC, pessimistic one-dose duration	100%	20 years	80%	-83.6% (-88.6% to -79.4%)	0.36 (0.24 to 0.49)	0.03 (0.00 to 0.04)	108.7% (101.0% to 117.5%)	85	107 (25 to 492)
2.b Sensitivity analyses of the secondary analysis									
**Adding MAC vaccination- low coverage:** Two-dose routine vaccination of 9-year-old girls + two- or one-dose MAC of 10-14-year-old girls									
• Two-dose MAC	100%	lifetime	50%	-84.6% (-89.0% to -81.8%)	0.37 (0.24 to 0.51)	0.03 (0.01 to 0.04)	110.8% (102.9% to 117.7%)	86	103 (66 to 259)
• One-dose MAC, non-inferior	100%	lifetime	50%	-84.6% (-89.0% to -81.8%)	0.37 (0.24 to 0.51)	0.03 (0.01 to 0.04)	110.8% (102.9% to 117.7%)	84	52 (33 to 130)
• One-dose MAC, pessimistic one-dose efficacy	85%	lifetime	50%	-83.9% (-88.1% to -81.5%)	0.36 (0.24 to 0.50)	0.03 (0.01 to 0.05)	109.1% (101.8% to 119.3%)	84	61 (30 to 154)
• One-dose MAC, pessimistic one-dose duration	100%	30 years	50%	-83.8% (-87.7% to -78.4%)	0.35 (0.23 to 0.50)	0.02 (0.01 to 0.04)	107.1% (101.9% to 111.6%)	84	79 (39 to 183)
• One-dose MAC, pessimistic one-dose duration	100%	20 years	50%	-83.8% (-87.6% to -80.9%)	0.35 (0.22 to 0.49)	0.02 (0.00 to 0.03)	105.4% (100.7% to 109.0%)	84	100 (44 to 229)

	Vaccine efficacy	Duration of protection	Vaccination coverage	Change in cervical cancer incidence after 100 years (%)<br><br>Mean (80% UI[+])	Averted cervical cancers (millions)<br><br>Mean (80% UI)	Difference in averted cervical cancers vs ref (millions)<br><br>Mean (80% UI)	Percentage of averted cervical cancers vs ref (%)<br><br>Mean (80% UI)	Total number of doses (millions)<br><br>Mean (80% UI)	NNV[¥]<br><br>Mean (80% UI)
**UGANDA**									
**1. Main analysis**									
1.a Base case analysis of the main analysis									
**Comparator:**<br>Two-dose routine vaccination of 9-year-old girls + two-dose MAC[*] vaccination of 10-14-years old girls	100%	lifetime	80%	-81.8%<br>(-85.1% to -78.0%)	2.25<br>(1.93 to 2.63)	Ref	Ref	145	65<br>(54 to 75)
**One-dose scenarios :**<br>One-dose routine vaccination of 9-year-old girls + one-dose MAC vaccination of 10-14-years old girls									
• Non-inferior one-dose vs two doses	100%	lifetime	80%	-81.8%<br>(-85.1% to -78.0%)	2.25<br>(1.93 to 2.63)	0<br>(0 to 0)	100%<br>(100% to 100%)	73	32<br>(27 to 38)
• Pessimistic one-dose efficacy	85%	lifetime	80%	-70.5%<br>(-74.7% to -67.3%)	1.93<br>(1.65 to 2.26)	-0.32<br>(-0.37 to -0.28)	85.7%<br>(84.5% to 87.2%)	73	38<br>(31 to 44)
• Pessimistic one-dose duration	100%	30 years	80%	-71.5%<br>(-78.5% to -68.5%)	1.99<br>(1.66 to 2.30)	-0.26<br>(-0.35 to -0.20)	88.4%<br>(85.9% to 91.3%)	73	37<br>(30 to 42)
• Pessimistic one-dose duration	100%	20 years	80%	-48.6%<br>(-53.5% to -43.8%)	1.33<br>(1.11 to 1.54)	-0.92<br>(-1.09 to -0.82)	59.0%<br>(54.7% to 62.4%)	73	55<br>(44 to 65)
**Mitigation strategy:**<br>One-dose routine vaccination for 5 years and switch to two doses + one-dose catch-up of 10-14-year-old	100%<br>100%	20 years<br>lifetime	80%<br>80%	-81.4%<br>(-85.4% to -79.0%)	2.19<br>(1.86 to 2.56)	-0.05<br>(-0.09 to -0.03)	97.5%<br>(96.3% to 98.6%)	142	65<br>(54 to 76)
1.b Sensitivity analyses of the main analysis									
**Low coverage :**<br>Two- or one-dose routine vaccination of 9-year-old girls + two- or one-dose MAC vaccination of 10-14-years old girls									
• Two-dose	100%	lifetime	40%	-43.8%<br>(-47.1% to -40.7%)	1.19<br>(1.00 to 1.38)	Ref	Ref	73	61<br>(49 to 72)

	Vaccine efficacy	Duration of protection	Vaccination coverage	Change in cervical cancer incidence after 100 years (%) Mean (80% UI[+])	Averted cervical cancers (millions) Mean (80% UI)	Difference in averted cervical cancers vs ref (millions) Mean (80% UI)	Percentage of averted cervical cancers vs ref (%) Mean (80% UI)	Total number of doses (millions) Mean (80% UI)	NNV[¥] Mean (80% UI)
• Non-inferior one-dose vs two doses	100%	lifetime	40%	-43.8% (-47.1% to -40.7%)	1.19 (1.00 to 1.38)	0 (0 to 0)	100% (100% to 100%)	36	31 (25 to 36)
• Pessimistic one-dose efficacy	85%	lifetime	40%	-37.0% (-42.1% to -32.0%)	1.00 (0.85 to 1.18)	-0.18 (-0.26 to -0.15)	84.5% (82.6% to 86.1%)	36	36 (30 to 42)
• Pessimistic one-dose duration	100%	30 years	40%	-38.3% (-42.8% to -35.4%)	1.02 (0.85 to 1.17)	-0.16 (-0.22 to -0.10)	86.3% (82.1% to 90.9%)	36	36 (29 to 42)
• Pessimistic one-dose duration	100%	20 years	40%	-25.9% (-31.2% to -21.7%)	0.69 (0.55 to 0.86)	-0.50 (-0.62 to -0.42)	58.0% (52.7% to 62.4%)	36	53 (42 to 64)
**High coverage (elimination target):** Two- or ne-dose routine vaccination of 9-year-old girls + two- or one-dose MAC vaccination of 10-14-years old girls									
• Two-dose	100%	lifetime	90%	-90.0% (-94.0% to -85.3%)	2.49 (2.13 to 2.91)	Ref	Ref	164	66 (54 to 76)
• Non-inferior one-dose vs two doses	100%	lifetime	90%	-90.0% (-94.0% to -85.3%)	2.49 (2.13 to 2.91)	0 (0 to 0)	100% (100% to 100%)	82	33 (27 to 38)
• Pessimistic one-dose efficacy	85%	lifetime	90%	-77.8% (-81.4% to -75.1%)	2.10 (1.81 to 2.46)	-0.39 (-0.45 to -0.33)	84.4% (83.2% to 85.1%)	82	39 (33 to 45)
• Pessimistic one-dose duration	100%	30 years	90%	-80.4% (-86.8% to -77.4%)	2.18 (1.82 to 2.53)	-0.31 (-0.39 to -0.24)	87.5% (85.2% to 90.4%)	82	38 (31 to 44)
• Pessimistic one-dose duration	100%	20 years	90%	-52.9% (-57.4% to -47.5%)	1.45 (1.19 to 1.71)	-1.04 (-1.26 to -0.94)	58.2% (53.9% to 61.2%)	82	56 (47 to 67)
**Switch from two- to one-dose vaccination program:** One-dose routine vaccination of 9-year-old girls after five years of two-dose vaccination									
• Non-inferior one-dose vs two doses	100%	lifetime	80%	-81.8% (-85.1% to -78.0%)	2.25 (1.93 to 2.63)	0 (0 to 0)	100% (100% to 100%)	78	35 (29 to 40)
• Pessimistic one-dose efficacy	85%	lifetime	80%	-70.1% (-73.4% to -66.6%)	1.97 (1.70 to 2.30)	-0.27 (-0.33 to -0.24)	87.8% (86.8% to 88.7%)	78	40 (33 to 46)
• Pessimistic one-dose duration	100%	30 years	80%	-71.8% (-76.8% to -69.4%)	2.03 (1.72 to 2.37)	-0.22 (-0.28 to -0.17)	90.3% (88.7% to 92.6%)	78	39 (32 to 45)
• Pessimistic one-dose duration	100%	20 years	80%	-48.0% (-51.8% to -46.0%)	1.46 (1.23 to 1.69)	-0.79 (-0.94 to -0.70)	64.7% (61.0% to 67.4%)	78	54 (44 to 64)

	Vaccine efficacy	Duration of protection	Vaccination coverage	Change in cervical cancer incidence after 100 years (%)<br>Mean (80% UI[+])	Averted cervical cancers (millions)<br>Mean (80% UI)	Difference in averted cervical cancers vs ref (millions)<br>Mean (80% UI)	Percentage of averted cervical cancers vs ref (%)<br>Mean (80% UI)	Total number of doses (millions)<br>Mean (80% UI)	NNV[¥]<br>Mean (80% UI)
**2. Secondary analysis**									
2.a Base case analysis of the secondary analysis									
**Comparator:**<br>Two-dose routine vaccination of 9-year-old girls	100%	lifetime	80%	-81.6%<br>(-86.2% to -77.0%)	2.15<br>(1.85 to 2.51)	Ref	Ref	140	Ref
**Adding MAC vaccination:**<br>Two-dose routine vaccination of 9-year-old girls<br>+ two- or one-dose MAC of 10-14-year-old girls									
• Two-dose MAC	100%	lifetime	80%	-81.8%<br>(-85.1% to -78.0%)	2.25<br>(1.93 to 2.63)	0.10<br>(0.08 to 0.12)	104.7%<br>(104.0% to 105.2%)	145	51<br>(41 to 64)
• One-dose MAC, non-inferior one-dose	100%	lifetime	80%	-81.8%<br>(-85.1% to -78.0%)	2.25<br>(1.93 to 2.63)	0.10<br>(0.08 to 0.12)	104.7%<br>(104.0% to 105.2%)	143	25<br>(20 to 32)
• One-dose MAC, pessimistic one-dose efficacy	85%	lifetime	80%	-81.2%<br>(-86.1% to -75.7%)	2.23<br>(1.91 to 2.62)	0.09<br>(0.06 to 0.11)	104.0%<br>(103.2% to 104.5%)	143	30<br>(22 to 38)
• One-dose MAC, pessimistic one-dose duration	100%	30 years	80%	-81.1%<br>(-85.1% to -77.2%)	2.24<br>(1.91 to 2.62)	0.09<br>(0.06 to 0.12)	104.2%<br>(103.2% to 104.7%)	143	28<br>(22 to 39)
• One-dose MAC, pessimistic one-dose duration	100%	20 years	80%	-81.2%<br>(-85.6% to -78.6%)	2.21<br>(1.89 to 2.59)	0.06<br>(0.04 to 0.08)	103.0%<br>(102.2% to 103.4%)	143	40<br>(31 to 56)
2.b Sensitivity analyses of the secondary analysis									
**Adding MAC vaccination- low coverage:**<br>Two-dose routine vaccination of 9-year-old girls<br>+ two- or one-dose MAC of 10-14-year-old girls									
• Two-dose MAC	100%	lifetime	50%	-81.4%<br>(-85.2% to -78.2%)	2.21<br>(1.89 to 2.59)	0.06<br>(0.03 to 0.08)	102.9%<br>(101.7% to 103.9%)	143	51<br>(38 to 91)
• One-dose MAC, non-inferior	100%	lifetime	50%	-81.4%<br>(-85.2% to -78.2%)	2.21<br>(1.89 to 2.59)	0.06<br>(0.03 to 0.08)	102.9%<br>(101.7% to 103.9%)	142	26<br>(19 to 45)
• One-dose MAC, pessimistic one-dose efficacy	85%	lifetime	50%	-81.1%<br>(-85.8% to -76.7%)	2.20<br>(1.88 to 2.58)	0.05<br>(0.03 to 0.07)	102.4%<br>(101.4% to 103.0%)	142	31<br>(23 to 46)
• One-dose MAC, pessimistic one-dose duration	100%	30 years	50%	-81.2%<br>(-85.5% to -78.5%)	2.20<br>(1.88 to 2.58)	0.06<br>(0.04 to 0.07)	102.6%<br>(101.9% to 103.2%)	142	29<br>(24 to 40)
• One-dose MAC, pessimistic one-dose duration	100%	20 years	50%	-81.1%<br>(-85.1% to -77.6%)	2.19<br>(1.88 to 2.57)	0.04<br>(0.02 to 0.05)	101.8%<br>(101.2% to 102.3%)	142	41<br>(31 to 68)

	Vaccine efficacy	Duration of protection	Vaccination coverage	Change in cervical cancer incidence after 100 years (%)<br><br>Mean (80% UI[+])	Averted cervical cancers (millions)<br><br>Mean (80% UI)	Difference in averted cervical cancers vs ref (millions)<br><br>Mean (80% UI)	Percentage of averted cervical cancers vs ref (%)<br><br>Mean (80% UI)	Total number of doses (millions)<br><br>Mean (80% UI)	NNV[¥]<br><br>Mean (80% UI)
**NIGERIA**									
**1. Main analysis**									
1.a Base case analysis of the main analysis									
**Comparator:**<br>Two-dose routine vaccination of 9-year-old girls + two-dose MAC[*] vaccination of 10-14-years old girls	100%	lifetime	80%	-76.1%<br>(-88.0% to -59.9%)	4.72<br>(3.43 to 5.76)	Ref	Ref	643	136<br>(105 to 185)
**One-dose scenarios :**<br>One-dose routine vaccination of 9-year-old girls + one-dose MAC vaccination of 10-14-years old girls									
• Non-inferior one-dose vs two doses	100%	lifetime	80%	-76.1%<br>(-88.0% to -59.9%)	4.72<br>(3.43 to 5.76)	0<br>(0 to 0)	100%<br>(100% to 100%)	321	68<br>(52 to 92)
• Pessimistic one-dose efficacy	85%	lifetime	80%	-65.2%<br>(-76.1% to -50.4%)	4.05<br>(2.92 to 4.95)	-0.67<br>(-0.98 to -0.47)	86.0%<br>(84.0% to 87.5%)	321	79<br>(62 to 106)
• Pessimistic one-dose duration	100%	30 years	80%	-71.4%<br>(-85.2% to -57.1%)	4.45<br>(3.22 to 5.52)	-0.27<br>(-0.40 to -0.16)	94.4%<br>(92.4% to 95.9%)	321	72<br>(55 to 97)
• Pessimistic one-dose duration	100%	20 years	80%	-51.5%<br>(-64.3% to -40.8%)	3.23<br>(2.44 to 4.06)	-1.49<br>(-1.95 to -1.03)	68.6%<br>(65.3% to 71.4%)	321	99<br>(77 to 131)
**Mitigation strategy:**<br>One-dose routine vaccination for 5 years and switch to two doses + one-dose catch-up of 10-14-year-old	100%<br>100%	20 years<br>lifetime	80%<br>80%	-76.3%<br>(-86.1% to -61.6%)	4.67<br>(3.40 to 5.66)	-0.06<br>(-0.12 to 0.00)	98.9%<br>(97.4% to 100.0%)	631	135<br>(104 to 183)
1.b Sensitivity analyses of the main analysis									
**Low coverage :**<br>Two- or one-dose routine vaccination of 9-year-old girls + two- or one-dose MAC vaccination of 10-14-years old girls									
• Two-dose	100%	lifetime	40%	-38.4%<br>(-46.8% to -31.0%)	2.44<br>(1.75 to 3.04)	Ref	Ref	321	132<br>(105 to 179)

	Vaccine efficacy	Duration of protection	Vaccination coverage	Change in cervical cancer incidence after 100 years (%) Mean (80% UI[+])	Averted cervical cancers (millions) Mean (80% UI)	Difference in averted cervical cancers vs ref (millions) Mean (80% UI)	Percentage of averted cervical cancers vs ref (%) Mean (80% UI)	Total number of doses (millions) Mean (80% UI)	NNV[¥] Mean (80% UI)
• Non-inferior one-dose vs two doses	100%	lifetime	40%	-38.4% (-46.8% to -31.0%)	2.44 (1.75 to 3.04)	0 (0 to 0)	100% (100% to 100%)	161	66 (52 to 90)
• Pessimistic one-dose efficacy	85%	lifetime	40%	-33.2% (-41.7% to -25.1%)	2.06 (1.49 to 2.57)	-0.39 (-0.48 to -0.24)	84.3% (81.5% to 86.3%)	161	78 (60 to 104)
• Pessimistic one-dose duration	100%	30 years	40%	-36.2% (-45.8% to -30.5%)	2.28 (1.63 to 2.88)	-0.17 (-0.31 to -0.11)	93.2% (90.7% to 95.7%)	161	71 (55 to 95)
• Pessimistic one-dose duration	100%	20 years	40%	-25.9% (-34.6% to -18.3%)	1.62 (1.05 to 2.05)	-0.82 (-1.12 to -0.52)	66.5% (63.3% to 71.0%)	161	99 (77 to 125)
**High coverage (elimination target) :** Two- or ne-dose routine vaccination of 9-year-old girls + two- or one-dose MAC vaccination of 10-14-years old girls									
• Two-dose	100%	lifetime	90%	-84.0% (-97.8% to -68.3%)	5.26 (3.81 to 6.37)	Ref	Ref	722	137 (107 to 188)
• Non-inferior one-dose vs two doses	100%	lifetime	90%	-84.0% (-97.8% to -68.3%)	5.26 (3.81 to 6.37)	0 (0 to 0)	100% (100% to 100%)	361	69 (53 to 94)
• Pessimistic one-dose efficacy	85%	lifetime	90%	-72.7% (-86.7% to -57.2%)	4.42 (3.20 to 5.38)	-0.84 (-1.12 to -0.63)	84.1% (81.5% to 86.0%)	361	82 (64 to 110)
• Pessimistic one-dose duration	100%	30 years	90%	-79.1% (-91.7% to -67.3%)	4.86 (3.55 to 5.91)	-0.40 (-0.55 to -0.28)	92.3% (89.7% to 93.9%)	361	74 (57 to 101)
• Pessimistic one-dose duration	100%	20 years	90%	-56.8% (-67.6% to -44.4%)	3.57 (2.67 to 4.41)	-1.69 (-2.18 to -1.15)	68.0% (65.0% to 70.9%)	361	101 (78 to 132)
**Switch from two- to one-dose vaccination program:** One-dose routine vaccination of 9-year-old girls after five years of two-dose vaccination									
• Non-inferior one-dose vs two doses	100%	lifetime	80%	-76.1% (-88.0% to -59.9%)	4.72 (3.43 to 5.76)	0 (0 to 0)	100% (100% to 100%)	345	73 (56 to 99)
• Pessimistic one-dose efficacy	85%	lifetime	80%	-64.3% (-78.2% to -50.6%)	4.13 (2.99 to 5.14)	-0.59 (-0.80 to -0.44)	87.6% (85.9% to 89.2%)	345	84 (65 to 111)
• Pessimistic one-dose duration	100%	30 years	80%	-70.9% (-83.3% to -56.8%)	4.48 (3.26 to 5.57)	-0.24 (-0.35 to -0.17)	94.9% (92.8% to 96.2%)	345	77 (59 to 104)
• Pessimistic one-dose duration	100%	20 years	80%	-51.0% (-61.1% to -40.0%)	3.43 (2.47 to 4.33)	-1.29 (-1.65 to -0.91)	72.7% (69.3% to 75.6%)	345	101 (77 to 135)

	Vaccine efficacy	Duration of protection	Vaccination coverage	Change in cervical cancer incidence after 100 years (%) Mean (80% UI+)	Averted cervical cancers (millions) Mean (80% UI)	Difference in averted cervical cancers vs ref (millions) Mean (80% UI)	Percentage of averted cervical cancers vs ref (%) Mean (80% UI)	Total number of doses (millions) Mean (80% UI)	NNV¥ Mean (80% UI)
**2. Secondary analysis**									
2.a Base case analysis of the secondary analysis									
**Comparator:** Two-dose routine vaccination of 9-year-old girls	100%	lifetime	80%	-76.1% (-88.7% to -62.8%)	4.49 (3.24 to 5.50)	Ref	Ref	620	Ref
**Adding MAC vaccination:** Two-dose routine vaccination of 9-year-old girls + two- or one-dose MAC of 10-14-year-old girls									
• Two-dose MAC	100%	lifetime	80%	-76.1% (-88.0% to -59.9%)	4.72 (3.43 to 5.76)	0.23 (0.14 to 0.29)	105.1% (103.8% to 106.4%)	643	97 (71 to 155)
• One-dose MAC, non-inferior one-dose	100%	lifetime	80%	-76.1% (-88.0% to -59.9%)	4.72 (3.43 to 5.76)	0.23 (0.14 to 0.29)	105.1% (103.8%-106.4%)	632	49 (35 to 77)
• One-dose MAC, pessimistic one-dose efficacy	85%	lifetime	80%	-75.8% (-89.1% to -61.9%)	4.70 (3.40 to 5.68)	0.21 (0.11 to 0.26)	104.8% (102.8% to 105.8%)	632	54 (41 to 101)
• One-dose MAC, pessimistic one-dose duration	100%	30 years	80%	-76.4% (-88.6% to -59.7%)	4.73 (3.43 to 5.72)	0.24 (0.13 to 0.31)	105.5% (103.7% to 106.0%)	632	46 (35 to 82)
• One-dose MAC, pessimistic one-dose duration	100%	20 years	80%	-75.9% (-89.4% to -62.1%)	4.68 (3.40 to 5.69)	0.19 (0.11 to 0.23)	104.4% (103.0% to 105.5%)	632	59 (45 to 91)
2.b Sensitivity analyses of the secondary analysis									
**Adding MAC vaccination- low coverage:** Two-dose routine vaccination of 9-year-old girls + two- or one-dose MAC of 10-14-year-old girls									
• Two-dose MAC	100%	lifetime	50%	-76.2% (-89.0% to -60.2%)	4.66 (3.38 to 5.63)	0.17 (0.08 to 0.22)	103.9% (102.1% to 104.1%)	634	84 (57 to 166)
• One-dose MAC, non-inferior	100%	lifetime	50%	-76.2% (-89.0% to -60.2%)	4.66 (3.38 to 5.63)	0.17 (0.08 to 0.22)	103.9% (102.1% to 104.1%)	627	42 (28 to 83)
• One-dose MAC, pessimistic one-dose efficacy	85%	lifetime	50%	-75.6% (-88.7% to -59.9%)	4.62 (3.36 to 5.64)	0.13 (0.04 to 0.16)	102.9% (101.2% to 103.4%)	627	55 (40 to 134)
• One-dose MAC, pessimistic one-dose duration	100%	30 years	50%	-75.8% (-87.8% to -61.0%)	4.64 (3.37 to 5.66)	0.15 (0.08 to 0.18)	103.6% (102.2% to 103.6%)	627	46 (37 to 88)
• One-dose MAC, pessimistic one-dose duration	100%	20 years	50%	-75.9% (-86.3% to -59.6%)	4.61 (3.32 to 5.59)	0.12 (0.05 to 0.16)	102.7% (101.0% to 103.5%)	627	59 (39 to 133)

+UI : uncertainty interval (80% UI: 10th -90th percentiles of the 20 parameter sets) and mean of the 20 parameter sets. ¥ To estimate the efficiency of routine vaccination, we calculated the NNVs of one- and two-dose routine vaccination strategies vs no vaccination. To estimate the efficiency of MAC vaccination, we calculated the incremental NNVs of one- and two-dose MAC vaccination strategies vs two-dose routine vaccination, in order to isolate the impact of MAC vaccination. * MAC: Multi-age cohort vaccination of 10-14-year-old girls during the first year of vaccination. Of note, uncertainty intervals should not be interpreted as confidence interval from a statistical point of view. Uncertainty intervals reflect uncertainty in model parameters and variability in sexual behaviour and HPV epidemiology within a country. To compare the results between vaccination strategies the uncertainty intervals around the following outcomes should be used: Difference in averted cervical cancers vs ref and Percentage of averted cervical cancers vs ref (%).

Figure S1: Waning of vaccine protection.
A) Waning of vaccine protection following a normal distribution (5-year standard deviation); B) Waning of vaccine protection following an exponential distribution.

A)

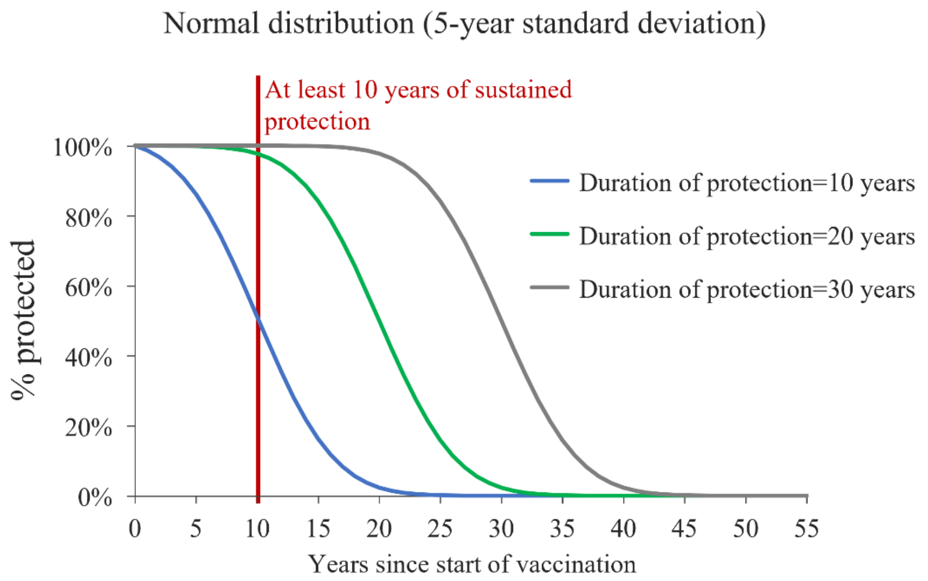

Given that studies have shown a sustained protection of at least 10 years,[4-7] as well as stable antibody titres over the entire period, it is implausible that the average one-dose duration of protection is 10 years or shorter. Based on the same data, it is unlikely that one-dose average duration of protection is shorter than 20 years. Therefore, in our analysis, we used a normal distribution of 20 years or 30 years, which allows to reproduce a stable vaccine efficacy for a set number of years before waning occurs.

B)

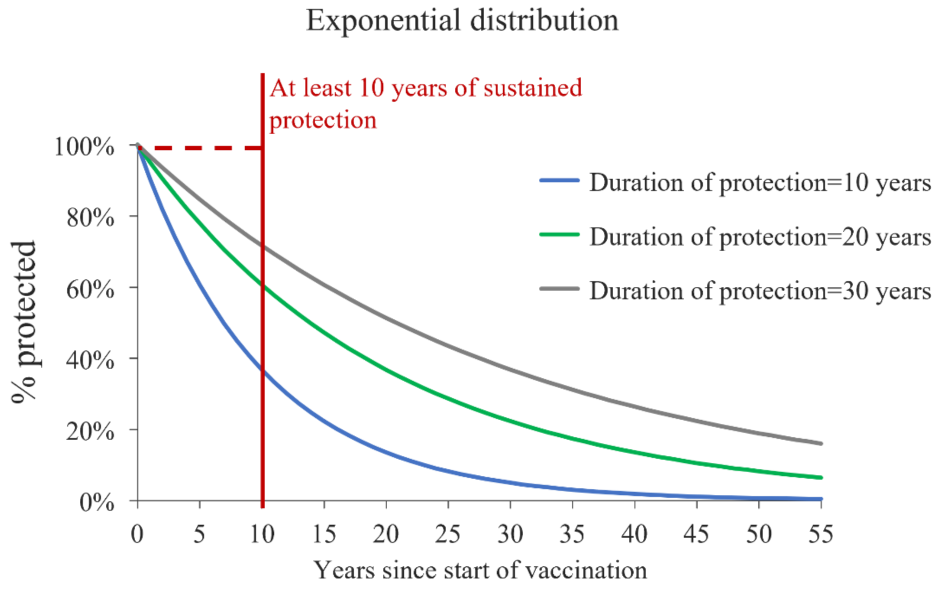

If waning of protection was constant (following an exponential distribution), we would already be seeing signs of waning.

Figure S2: Projected population-level impact of one- and two-dose routine vaccination of 9-year-old girls (with MAC vaccination of 10-14-year-old girls) assuming 40% vaccination coverage.

A) Change in cervical cancer incidence over time since start of vaccination (vs. no vaccination); B) Change in cervical cancer incidence after 100 years (vs. no vaccination); C) Averted cervical cancers over 100 years after start of vaccination (vs. no vaccination), in millions;); D) Difference in averted cervical cancers (vs. two-dose routine vaccination), in millions; E) Percentage of averted cervical cancers (vs. two-dose routine vaccination).

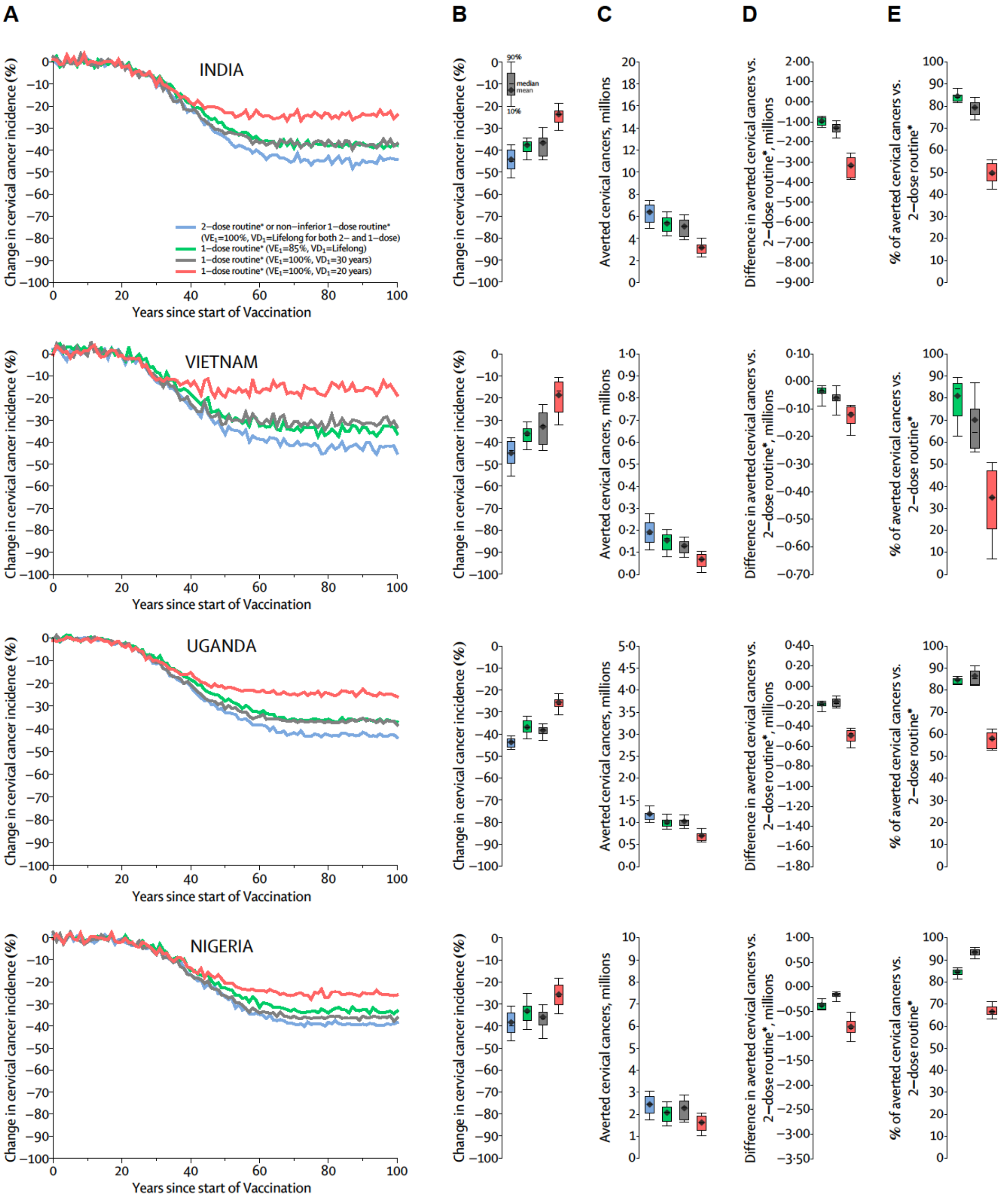

*Routine vaccination is always combined with MAC vaccination of 10-14-year-old girls (within the first year of vaccination, with the same number of doses and coverage as routine). Vaccination is assumed to start in 2023. Vaccination coverage for routine and MAC vaccination=40%. VE_i=vaccine efficacy of dose i. VD_i=vaccine duration of protection of dose i. In all scenarios: two-dose VE=100%, two-dose VD=life. For A, the lines represent the mean of the 20 parameter sets. For B, C, D, and E: boxplots: lower and upper limits: 10th and 90th percentiles of the 20 parameter sets, box: 25th and 75th percentiles of the 20 parameter sets, line: median of the 20 parameter sets, diamond: mean of the 20 parameter sets. Of note, uncertainty intervals should not be interpreted as confidence interval from a statistical point of view. Uncertainty intervals reflect uncertainty in model parameters and variability in HPV epidemiology within a country. To compare the results between vaccination strategies, the uncertainty intervals in figures D and E should be used.

Figure S3: Projected population-level impact of one- and two-dose routine vaccination of 9-year-old girls (with MAC vaccination of 10-14-year-old girls) assuming 90% vaccination coverage.

A) Change in cervical cancer incidence over time since start of vaccination (vs. no vaccination); B) Change in cervical cancer incidence after 100 years (vs. no vaccination); C) Averted cervical cancers over 100 years after start of vaccination (vs. no vaccination), in millions; D) Difference in averted cervical cancers (vs. two-dose routine vaccination), in millions; E) Percentage of averted cervical cancers (vs. two-dose routine vaccination).

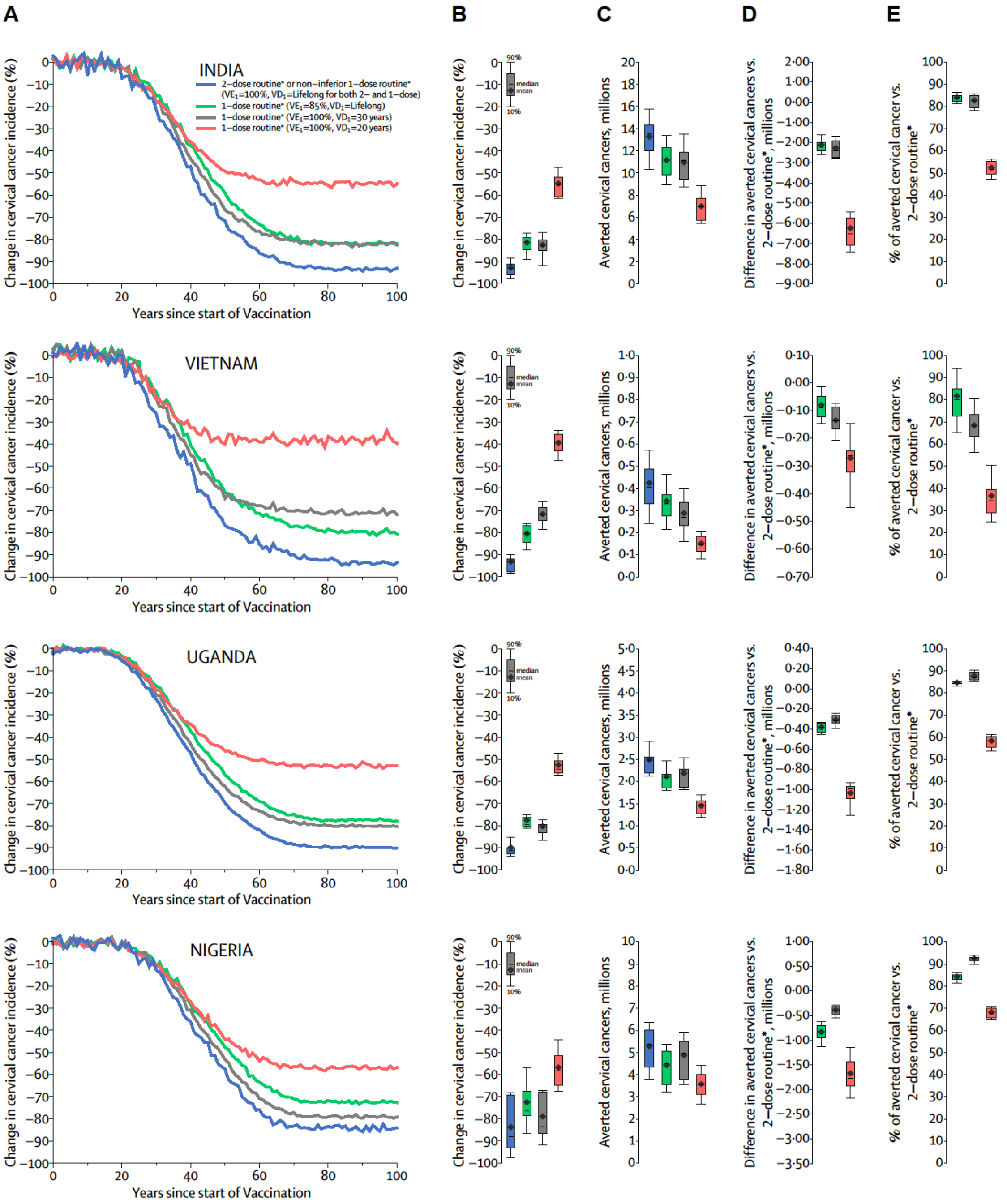

*Routine vaccination is always combined with MAC vaccination of 10-14-year-old girls (within the first year of vaccination, with the same number of doses and coverage as routine). Vaccination is assumed to start in 2023. Vaccination coverage for routine and MAC vaccination=90%. VE_i=vaccine efficacy of dose i. VD_i=vaccine duration of protection of dose i. In all scenarios: two-dose VE=100%, two-dose VD=life. For A, the lines represent the mean of the 20 parameter sets. For B, C, D, and E: boxplots: lower and upper limits: 10th and 90th percentiles of the 20 parameter sets, box: 25th and 75th percentiles of the 20 parameter sets, line: median of the 20 parameter sets, diamond: mean of the 20 parameter sets. Of note, uncertainty intervals should not be interpreted as confidence interval from a statistical point of view. Uncertainty intervals reflect uncertainty in model parameters and variability in HPV epidemiology within a country. To compare the results between vaccination strategies, the uncertainty intervals in figures D and E should be used.

Figure S4: Projected population-level impact of switching from two- to one-dose routine vaccination of 9-year-old girls after 5 years, assuming 80% vaccination coverage.

A) Change in cervical cancer incidence over time since start of vaccination (vs. no vaccination); B) Change in cervical cancer incidence after 100 years (vs. no vaccination); C) Averted cervical cancers over 100 years after start of vaccination (vs. no vaccination), in millions; D) Difference in averted cervical cancers (vs. two-dose routine vaccination), in millions; E) Percentage of averted cervical cancers (vs. two-dose routine vaccination).

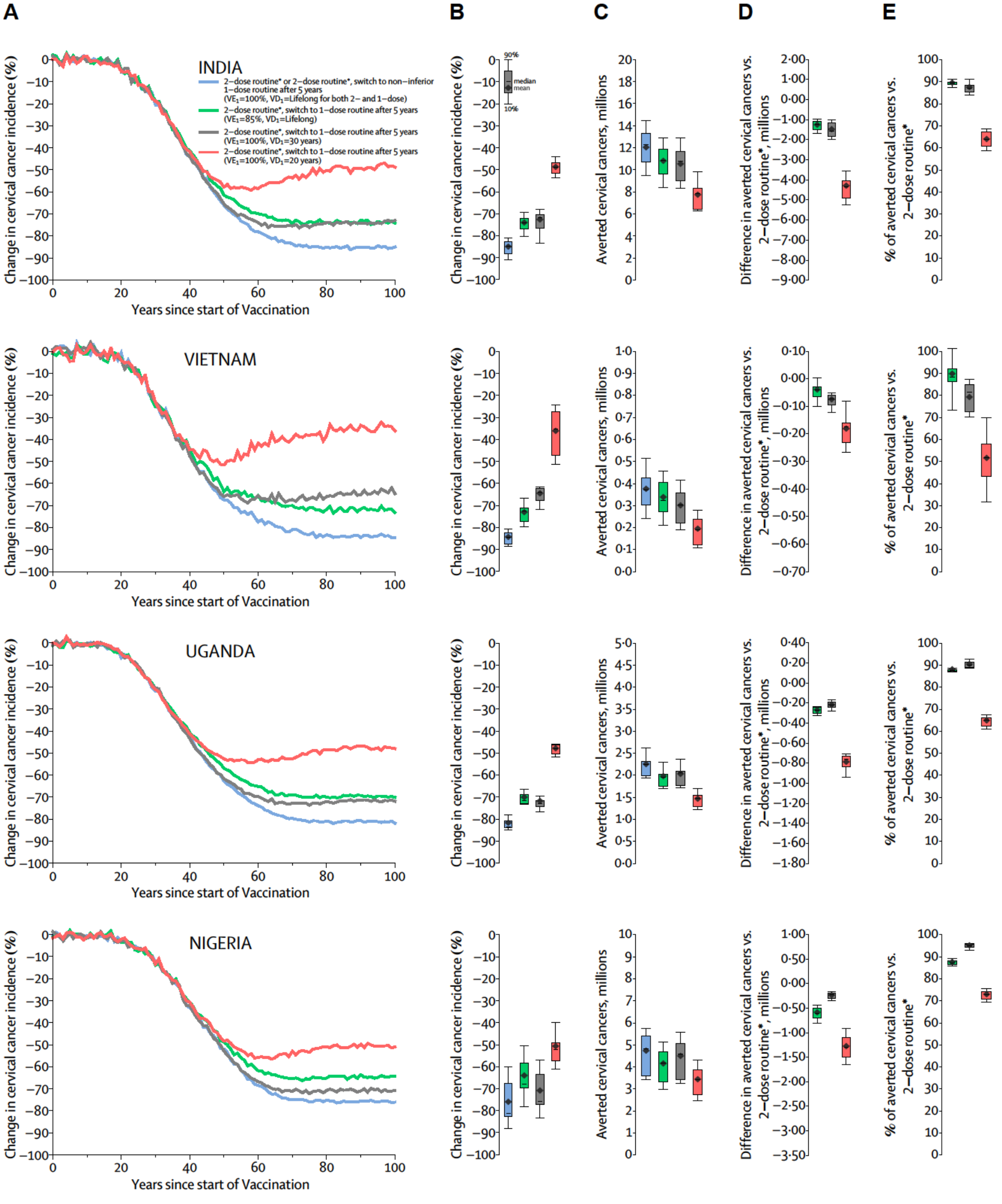

*Routine vaccination is always combined with MAC vaccination of 10-14-year-old girls (within the first year of vaccination, with the same number of doses and coverage as routine). Vaccination is assumed to start in 2023. Vaccination coverage for routine and MAC vaccination=80%. VE_i=vaccine efficacy of dose i. VD_i=vaccine duration of protection of dose i. In all scenarios: two-dose VE=100%, two-dose VD=life. For A, the lines represent the mean of the 20 parameter sets. For B, C, D, and E: boxplots: lower and upper limits: 10th and 90th percentiles of the 20 parameter sets, box: 25th and 75th percentiles of the 20 parameter sets, line: median of the 20 parameter sets, diamond: mean of the 20 parameter sets. Of note, uncertainty intervals should not be interpreted as confidence interval from a statistical point of view. Uncertainty intervals reflect uncertainty in model parameters and variability in HPV epidemiology within a country. To compare the results between vaccination strategies, the uncertainty intervals in figures D and E should be used.

Figure S5: Projected population-level impact of one- and two-dose routine vaccination of 9-year-old girls (with MAC vaccination of 10-14-year-old girls) assuming 80% vaccination coverage for one-dose scenarios and 40% vaccination coverage for two-dose scenario.

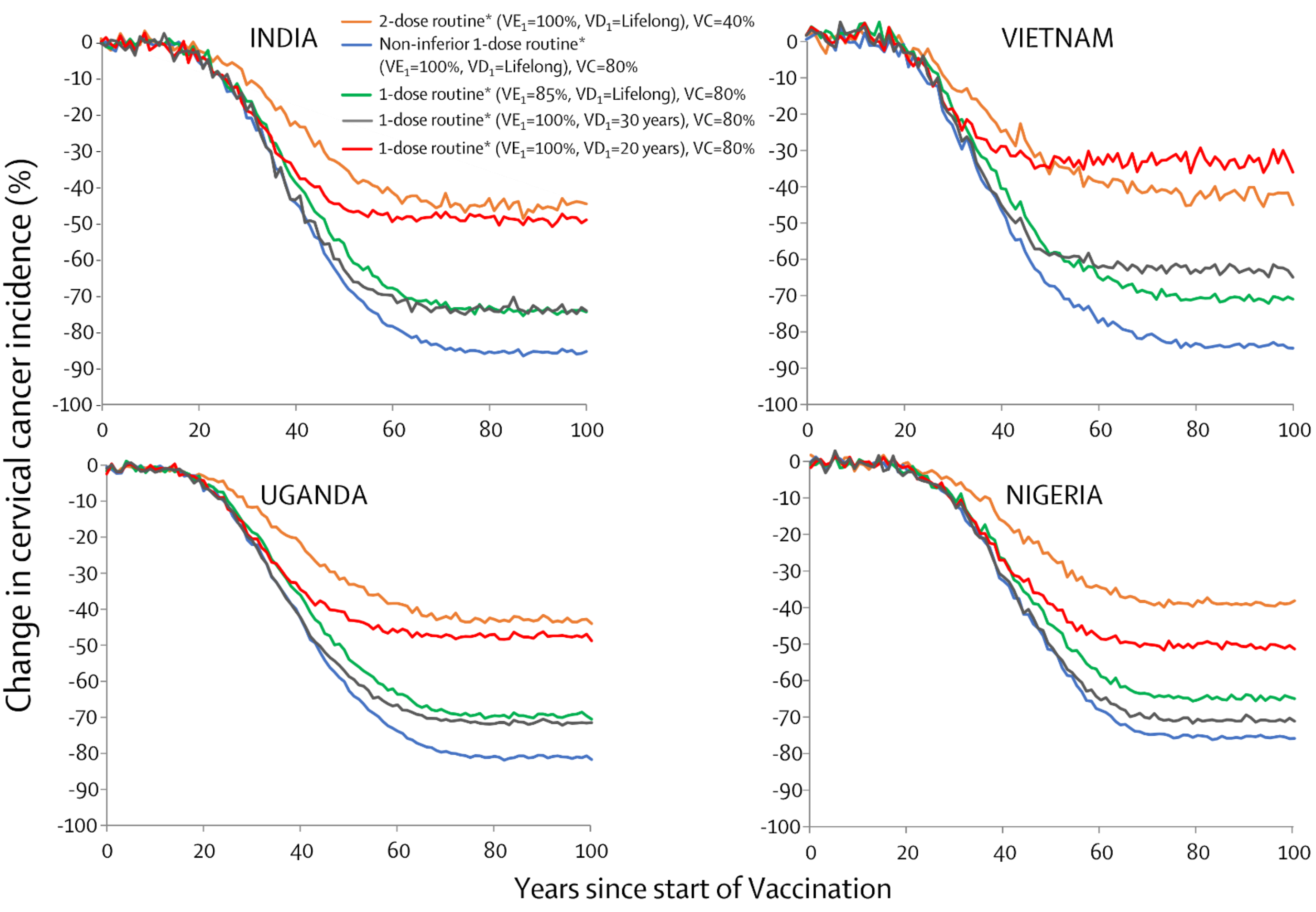

*Routine vaccination is always combined with MAC vaccination of 10-14-year-old girls (within the first year of vaccination, with the same number of doses and coverage as routine). Vaccination is assumed to start in 2023. VC=vaccination coverage. VE_i=vaccine efficacy of dose i. VD_i=vaccine duration of protection of dose i. In all scenarios: two-dose VE=100%, two-dose VD=life. The lines represent the mean of the 20 parameter sets.

Figure S6: Projected population-level impact of one- and two-dose MAC vaccination of 10-14-year-old girls (with two-dose routine vaccination of 9-year-old girls) assuming 80% MAC vaccination coverage.

A) Change in cervical cancer incidence over time since start of vaccination (vs. no vaccination); B) Change in cervical cancer incidence after 100 years (vs. no vaccination); C) Averted cervical cancers over 100 years after start of vaccination (vs. no vaccination), in millions; D) Difference in averted cervical cancers (vs. two-dose routine vaccination), in millions; E) Percentage of averted cervical cancers (vs. two-dose routine vaccination).

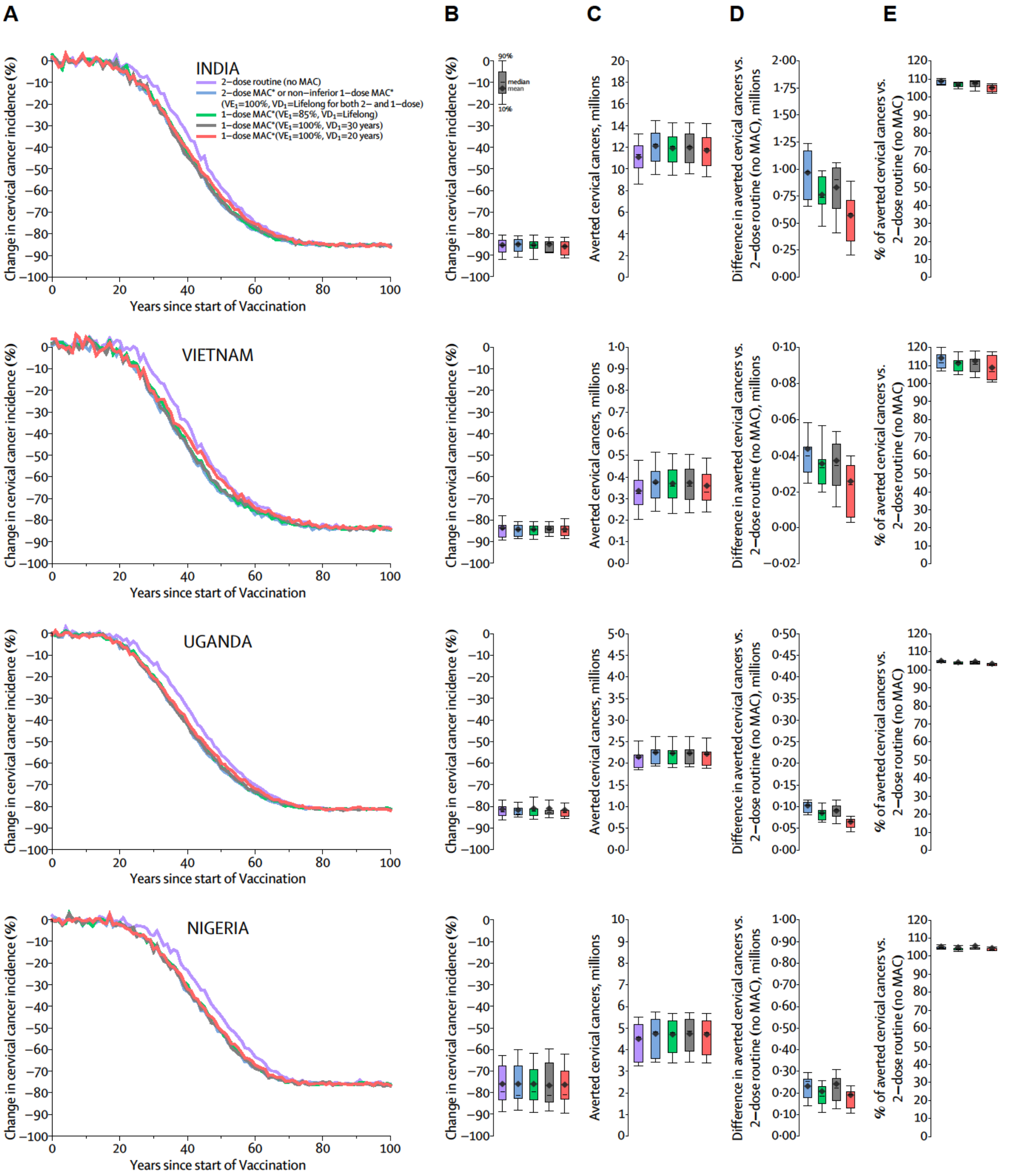

*MAC vaccination (within the first year of vaccination) is always combined with two-dose routine vaccination of 9-year-old girls. Vaccination is assumed to start in 2023. Vaccination coverage for routine and MAC vaccination=80%. VE_i=vaccine efficacy of dose i. VD_i=vaccine duration of protection of dose i. In all scenarios: two-dose VE=100%, two-dose VD=life. For A, the lines represent the mean of the 20 parameter sets. For B, C, D, and E: boxplots: lower and upper limits: 10th and 90th percentiles of the 20 parameter sets, box: 25th and 75th percentiles of the 20 parameter sets, line: median of the 20 parameter sets, diamond: mean of the 20 parameter sets. Of note, uncertainty intervals should not be interpreted as confidence interval from a statistical point of view. Uncertainty intervals reflect uncertainty in model parameters and variability in HPV epidemiology within a country. To compare the results between vaccination strategies, the uncertainty intervals in figures D and E should be used.

Figure S7: Projected population-level impact of one- and two-dose MAC vaccination of 10-14-year-old girls (with two-dose routine vaccination of 9-year-old girls) assuming 50% MAC vaccination coverage.

A) Change in cervical cancer incidence over time since start of vaccination (vs. no vaccination); B) Change in cervical cancer incidence after 100 years (vs. no vaccination); C) Averted cervical cancers over 100 years after start of vaccination (vs. no vaccination), in millions; D) Difference in averted cervical cancers (vs. two-dose routine vaccination), in millions; E) Percentage of averted cervical cancers (vs. two-dose routine vaccination).

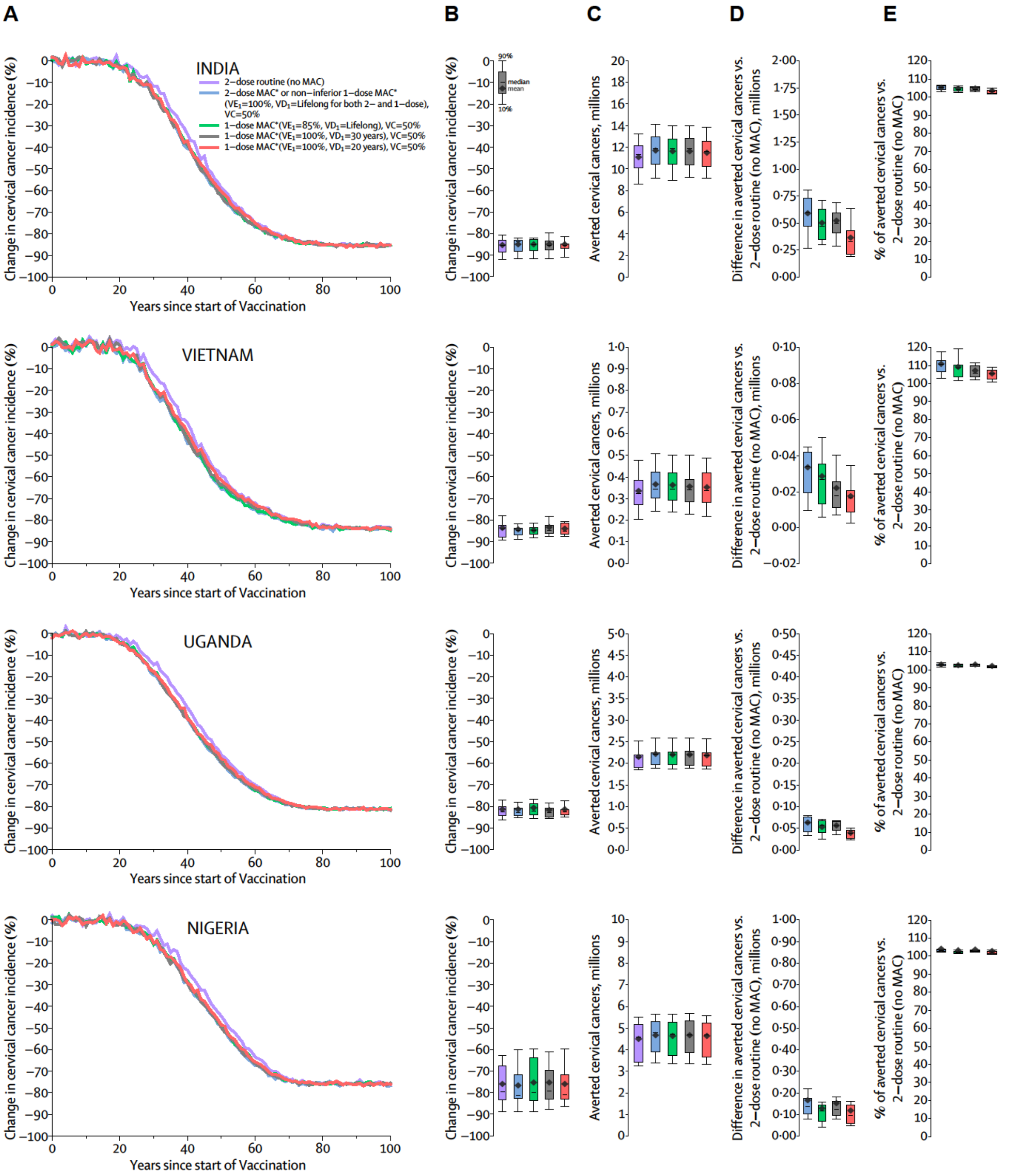

*MAC vaccination (within the first year of vaccination) is always combined with two-dose routine vaccination of 9-year-old girls. Vaccination is assumed to start in 2023. VC=vaccination coverage. VC for routine=80% and VC for MAC=50%. VE_i=vaccine efficacy of dose i. VD_i=vaccine duration of protection of dose i. In all scenarios: two-dose VE=100%, two-dose VD=life. For A, the lines represent the mean of the 20 parameter sets. For B, C, D, and E: boxplots: lower and upper limits: 10th and 90th percentiles of the 20 parameter sets, box: 25th and 75th percentiles of the 20 parameter sets, line: median of the 20 parameter sets, diamond: mean of the 20 parameter sets. Of note, uncertainty intervals should not be interpreted as confidence interval from a statistical point of view. Uncertainty intervals reflect uncertainty in model parameters and variability in HPV epidemiology within a country. To compare the results between vaccination strategies, the uncertainty intervals in figures D and E should be used.

Figure S8: Number of doses needed to prevent one cervical cancer (NNV) through one- and two-dose MAC vaccination.

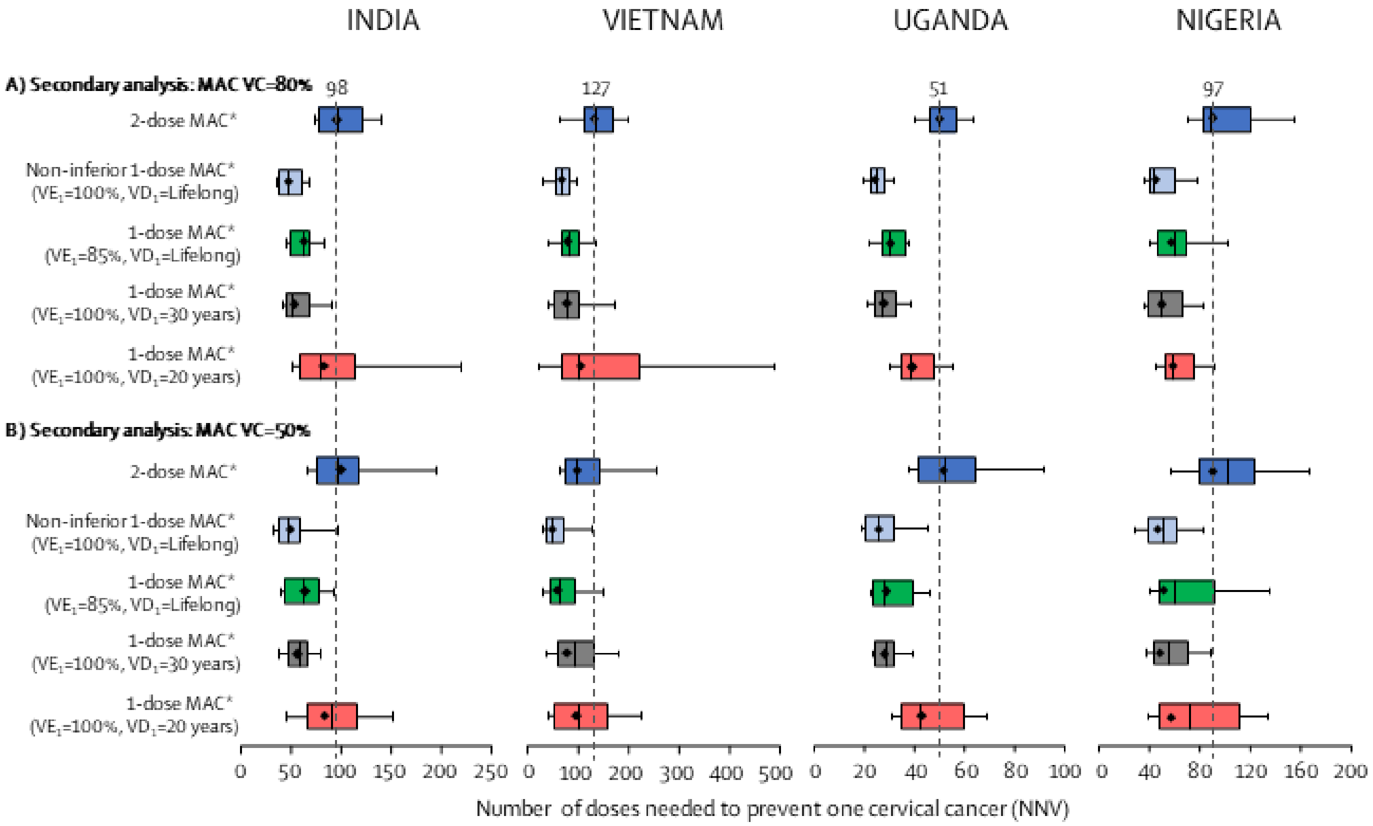

*MAC vaccination (within the first year of vaccination) is always combined with two-dose routine of 9-year-old girls. Vaccination is assumed to start in 2023. VC=vaccination coverage. VC is the same in routine and MAC vaccination. VE_i=vaccine efficacy of dose i. VD_i=vaccine duration of protection of dose i. In all scenarios: two-dose VE=100% and two-dose VD=life.Boxplots: lower and upper limits: 10th and 90th percentiles of the 20 parameter sets, box: 25th and 75th percentiles of the 20 parameter sets, line: median of the 20 parameter sets, diamond: mean of the 20 parameter sets. Of note, uncertainty intervals should not be interpreted as confidence interval from a statistical point of view. Uncertainty intervals reflect uncertainty in model parameters and variability in HPV epidemiology within a country.

Figure S9: HPV-16 infection in women over time, mean age at infection, and mean age at cervical cancer.

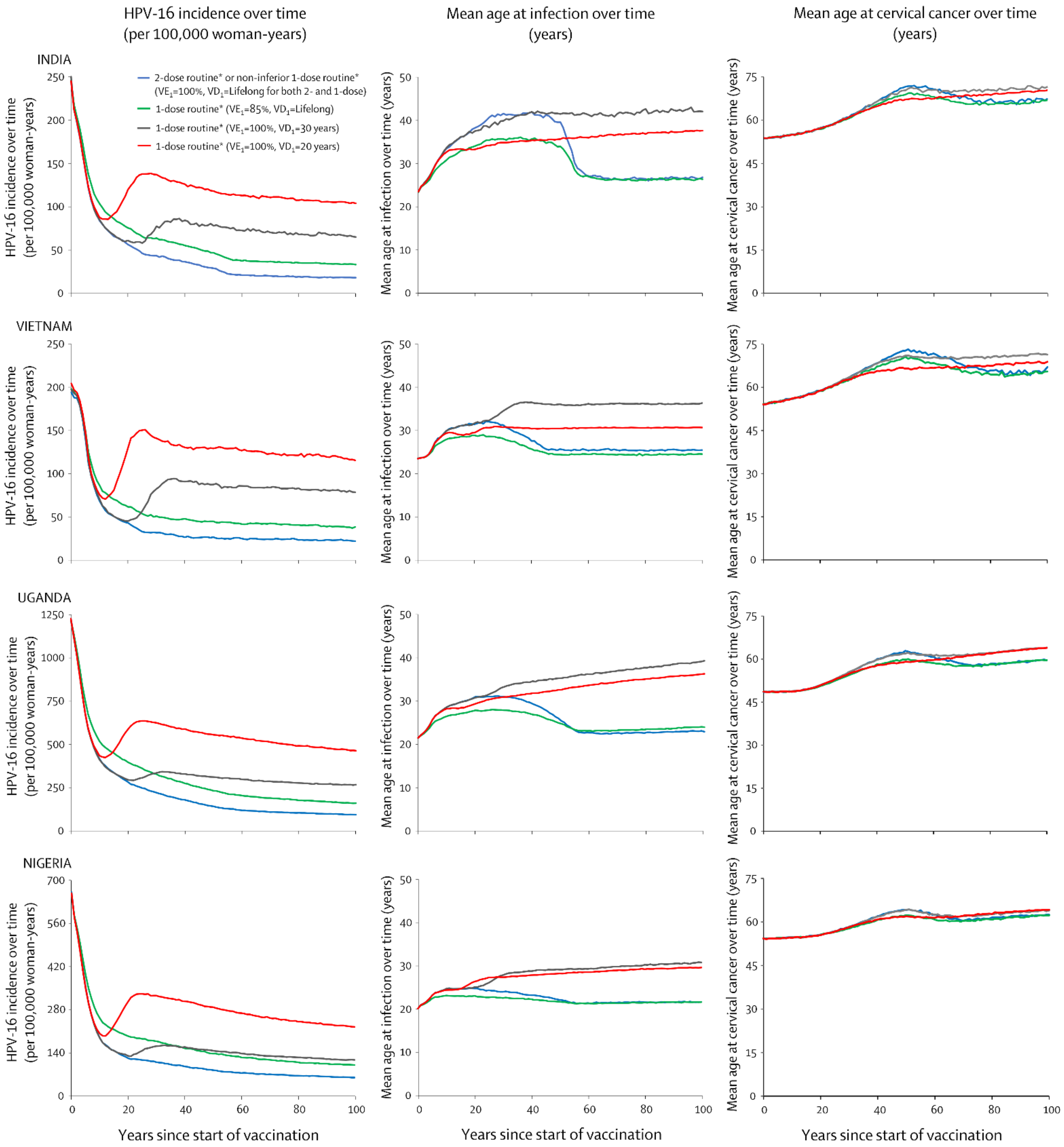

*Routine vaccination is always combined with MAC vaccination of 10-14-year-old girls (within the first year of vaccination, with the same number of doses and coverage as routine). Vaccination is assumed to start in 2023. Vaccination coverage for routine and MAC=80%. VE_i=vaccine efficacy of dose i. VD_i=vaccine duration of protection of dose i. In all scenarios: two-dose VE=100%, two-dose VD=life. The lines represent the mean of the 20 parameter sets.

Estimation of country-specific population size between 2100-2123

The age-stratified population for all countries between 2023 and 2100 were taken from United Nations World Population Prospects: The 2022 Revision (using the medium variant projections; medium-fertility assumption, normal mortality and normal international migration).[8] Because the model projections of cervical cancer cases averted were until 2123 and population data were only available up to 2100, we extrapolated the United Nations World Population from 2100 to 2123.

To do this, first, we defined a population matrix (Pa,y) representing the number of people of age group "a" (five-year age groups) at year "y" (between 2000-2100). Second, we defined the effective survival rates ((Sa,y)= (Pa+1,y)/ (Pa,y-5)) as the ratio of the population of the subsequent age group over the population of the age group five years before. The effective birth rate ((B0-4,y)=(P0-4,y)) was defined as the 0-4 years old population. As survival and birth rates oscillate over time with different periods, we used Fourier analysis in the extrapolation process. The extrapolation of survival and birth rates after 2100 were performed in three steps: 1) for each age group, we removed the secular trend using a least-squares linear fit; 2) we performed a fast Fourier transform (FFT) and find local maxima in the power spectrum (dominant oscillatory components that have particular frequencies) that allowed us to define a least-squares fit (which is the sum of cosine functions representing each particular dominant frequency); and 3) we re-added the secular trend that was previously removed to these oscillatory components to get the full extrapolation results. Using this method, we estimated the effective survival rates and the birth rate for years 2100 onwards for all age groups and countries. To get the projections for the population for years 2101 to 2123, we used the birth rates and the effective survival rates ((P5-9,y)=(B0-4,y-5)·(S0-4,y)). Then, subsequent age group populations were obtained iteratively as ((Pa+1,y)=(Pa,y-5)·(Sa,y)).

References

1. Canfell K, Kim JJ, Kulasingam S, et al. HPV-FRAME: A consensus statement and quality framework for modelled evaluations of HPV-related cancer control. *Papillomavirus Res*. 2019;8:100184.
2. Brisson M, Kim JJ, Canfell K, et al. Impact of HPV vaccination and cervical screening on cervical cancer elimination: a comparative modelling analysis in 78 low-income and lower-middle-income countries. *Lancet* 2020; 395(10224): 575-90.
3. Canfell K, Kim JJ, Brisson M, et al. Mortality impact of achieving WHO cervical cancer elimination targets: a comparative modelling analysis in 78 low-income and lower-middle-income countries. *Lancet* 2020; 395(10224): 591-603.
4. Basu P, Malvi SG, Joshi S, et al. Vaccine efficacy against persistent human papillomavirus (HPV) 16/18 infection at 10 years after one, two, and three doses of quadrivalent HPV vaccine in girls in India: a multicentre, prospective, cohort study. *The Lancet Oncology* 2021; 22(11): 1518-29.
5. Basu P, Bhatla N, Muwonge R, et al. Multicentric cohort study to compare the long-term efficacy of a single-dose of 4-valent vaccine compared to two- and three-dose in 10-18 Yr old females in India. IPVC 2023, Washington DC April 17-21.
6. Kreimer AR, Sampson JN, Porras C, et al. Evaluation of Durability of a Single Dose of the Bivalent HPV Vaccine: The CVT Trial. *Journal of the National Cancer Institute* 2020; 112(10): 1038-46.
7. Romero B, Herrero R, Porras C, et al. Durability of HPV-16/18 antibodies 16 years after a single dose of the bivalent vaccine: The Costa Rica HPV Vaccine Trial. IPVC 2023, Washington DC April 17-21.
8. United Nations. World Population Prospects. Available at: https://population.un.org/wpp/Download/Standard/Population/. Accessed March 22, 2022.
